# Beyond Fetal Immunity: A Systematic Review and Meta-Analysis of the Association Between Antenatal Corticosteroids and Retinopathy of Prematurity

**DOI:** 10.3389/fphar.2022.759742

**Published:** 2022-01-28

**Authors:** Yue Zeng, Ge Ge, Chunyan Lei, Meixia Zhang

**Affiliations:** ^1^ Department of Ophthalmology, West China Hospital, Sichuan University, Chengdu, China; ^2^ Research Laboratory of Macular Disease, West China Hospital, Sichuan University, Chengdu, China

**Keywords:** antenatal corticosteroids, retinopathy of prematurity, fetal immunity, preterm birth, meta-analysis, systematic review

## Abstract

**Background:** Retinopathy of prematurity (ROP) is a major cause of childhood blindness. Antenatal corticosteroids (ACS) exposure is known to ameliorate the risk of and mortality of neonatal morbidities. However, the effect of ACS on ROP development is currently unknown. We conducted a meta-analysis with up-to-date evidence to assess the association between ACS exposure and the development of ROP in at-risk preterm infants.

**Methods:** PubMed, EMBASE, Scopus, Web of Science, and the Cochrane Library were systematically searched from inception to May 2021, supplemented with manual search from reference lists. Studies with a control group reporting ROP rate in ACS-exposed infants were included. Pooled odds ratios (ORs) and 95% confidence intervals (CIs) were calculated utilizing a random-effects model. The Newcastle-Ottawa Scale was used for assessment of risk of bias in the included studies. Meta-regressions were performed to explore the predictive role of confounders for between-study variance.

**Results:** A total of 63 studies, involving 196,264 infants, were included. Meta-analysis showed ACS exposure was not associated with ROP occurrence (uOR 0.92, 95% CI 0.80–1.07; aOR 0.87, 95% CI 0.7–1.08). Results from extremely immature subgroups revealed significant reduced risks of ROP occurrence in ACS-exposed infants. ACS exposure was associated with significantly lower odds of ROP progression in adjusted analysis (aOR 0.48, 95% CI 0.26–0.89) instead of unadjusted analysis (uOR 0.86, 95% CI 0.68–1.08). Meta-regression showed birth weight and patent ductus arteriosus of the cohort were associated with ROP occurrence, sample size and study design strongly associated with ROP progression in ACS-exposed infants.

**Conclusion:** ACS treatment may decrease, but not prevent, the severity of ROP. Findings from severe ROP should be interpreted with caution owing to limited studies and the possibility of false-positive results. Considering the particular benefits in extremely immature infants, we recommend routine usage of ACS in mothers with threatened delivery to this particular birth cohort to prevent ROP occurrence. Future studies adjusting for major confounders are warranted to mitigate risk of bias in such observational evidence.

**Systematic Review Registration:** (https://www.crd.york.ac.uk/prospero/display_record.php?RecordID=270250), identifier (CRD42021270250)

## Introduction

Retinopathy of prematurity (ROP) is a major cause of childhood blindness associated with aberrant development of retinal vasculature in preterm infants (Hartnett, 2017). An estimation of 20,000 preterm babies is blinded from ROP annually, more than half of whom were born in middle-income regions (Blencowe et al., 2013). ROP, a biphasic disease, is initiated with blunted retinal vascular growth in the setting of hyperoxia (phase I), followed by abnormal retinal neovascularization in response to hypoxia-induced intraocular growth factors such as vascular endothelial growth factor (VEGF) (phase II) (Smith et al., 2013). Currently, postnatal factors interrupting normal retinal angiogenesis, such as supplemental oxygen therapy, have been well-recognized to be associated with ROP (Kim et al., 2018). However, little is known concerning prenatal factors related to ROP. A systemic review has suggested a prenatal “pre-phase” of ROP sensitizes the retina to in-utero inflammatory factors and subsequently triggers dysregulated angiogenesis (Lee and Dammann, 2012). Recently growing evidence shows antenatal exposure of inflammation increase the risk of ROP, which points to the possible involvement of immune pathway in ROP etiology (Dammann et al., 2009; Deliyanti et al., 2017; Villamor-Martinez et al., 2018). In this respect, prenatal factors interfering with fetal immune system might be a novel line for the pathology and prevention of ROP.

Antenatal corticosteroids (ACS), first introduced in 1972 (Liggins and Howie, 1972), are now recommended for mothers in anticipation of preterm delivery between 24–34 weeks’ gestation (Committee on Obstetric Practice, 2017). Those steroids easily reach the fetus and target the ubiquitously-expressed glucocorticoid receptors (GR), which may therefore affect the fetal immune system (Kemp et al., 2016). A single course of ACS reduces the rates of neonatal death, respiratory distress syndrome (RDS), necrotizing enterocolitis (NEC), intraventricular hemorrhage (IVH), and sepsis in preterm infants (Chawla et al., 2016; Roberts et al., 2017). Despite that, their effect on ROP has been debated for decades. In 1997, Console and others first directly address the association between ACS and ROP (Console et al., 1997). Since then, emerging evidence have demonstrated either decreased (van Sorge et al., 2014; Ying et al., 2019) or unaltered (Dammann et al., 2009; Eriksson et al., 2009) risk of ROP in ACS-exposed infants. Some studies also noted ACS-exposed babies may less likely to fall into severe ROP category (Maini et al., 2014). Recent meta-analysis by Yim et al. included 28 studies and concluded ACS was significantly associated with lower odds of both ROP occurrence and progression (Yim et al., 2018). However, those results are unstable according to the sensitivity analysis, partly due to the presence of important confounders.

After meta-analysis by Yim et al. have been published, a substantial number of relevant studies have been published. Some of them are large-scale cohort studies of high methodological quality. We therefore conducted an updated meta-analysis to examine the association between ACS exposure and ROP development, as well as the role of potential confounders.

## Materials and Methods

This meta-analysis was performed according to Preferred Reporting Items for Systematic Reviews and Meta-Analyses (PRISMA) guidelines (Moher et al., 2009). A PRISMA Checklist was presented in Supplementary Table S5. The protocol for this systematic review was previously registered on PROSPERO and can be accessed at http://www.crd.york.ac.uk/PROSPERO/, ID = CRD42021270250).

### Search Strategy

A comprehensive search of related studies was conducted in PubMed, EMBASE, Scopus, Web of Science, and the Cochrane Library from inception to May 2021. The following keywords were used as search terms: (“Retinopathy of Prematurity” OR “ROP” OR “Prematurity Retinopathy” OR “Retrolental Fibroplasia*” OR “Prematurity Retinopathies”) AND (“Steroid*” OR “Cortico*” OR “Betamethasone” OR “Dexamethasone”). Search strategy for other databases was adapted from the initial PubMed strategy. The detailed search strategies were listed in Supplementary Table S1. Additional strategy included a manual search from reference lists of all retrieved review papers and key articles.

### Eligibility Criteria

Studies were included if they met all of the following criteria: 1) original observational studies (cohort or case-control studies) or randomized controlled trials (RCTs). 2) reported ROP outcome of children whose mothers were administrated any type of ACS treatment regimen, including betamethasone, and dexamethasone, complete, and partial courses. 3) evaluated the outcome with odds ratios (ORs) and their confidence intervals (CIs), or with raw data provided for calculation. 4) studies reported in English or Chinese. The exclusion criteria were: 1) animal studies, reviews, comments, case reports, and conference abstracts. 2) Studies with no control arm or insufficient data. 3) overlapping cohorts from the same database or reporting repeated outcomes (study with more comprehensive data was included).

### Study Selection and Data Extraction

The author (YZ) searched the aforementioned database and assimilated an original list of literature. Two reviewers (YZ and GG) screened all retrieved records and subsequently extracted data using a standardized form independently. Discrepancies were resolved by checking the primary article and consulting a third reviewer (CL). Data were collected as follows: First author, year of publication, country of study, study design, inclusion criteria, type and course of steroid administration, sample size, association results (calculated odds ratio and 95% confidential interval), adjusted factors. Data based on different doses (complete or partial) and types of steroids (betamethasone or dexamethasone) was extracted separately for subgroup analysis. A complete or optimal course of antenatal steroid use was defined if 24 mg betamethasone (12 mg, two doses at 24-h intervals) or dexamethasone (6 mg, four doses at 12-h intervals) were prenatally administrated to mothers with threatened preterm labor. Severe ROP was variably defined as stage 3 and above, threshold ROP, type 1 ROP, or ROP requiring treatment (Early Treatment For Retinopathy Of Prematurity Cooperative Group, 2003; Chiang et al., 2021).

### Quality Assessment

Two reviewers (YZ and GG) independently evaluated the methodological quality of each study using the Newcastle-Ottawa Scale (Wells et al., 2011). This scale consists of 3 aspects: selection (0–4 points), comparability (0–2 points), and ascertainment of outcome (0–3 points). Studies were considered as satisfactory quality if they scored 5 and above out of 9. Any discrepancies were resolved through discussion or referred to a third reviewer (CL).

### Statistical Analysis

Stata 16.0 software (StataCorp LP, College Station, TX, United States) was used to combine and analyze the outcomes. To correspond to the majority of studies, individual studies with multiple adjusted ORs stratified by different types of steroids or number of embryos were pooled using the method by Hamling et al. to obtain combined risk for the whole population (Hamling et al., 2008). For subgroups receiving single and multiple courses of ACS, we only selected data for one course of steroid. When the data of GA and BW were given as the median (range or interquartile space), they were converted to the mean and SD using the statistical methods provided by Luo et al. (2018).

Due to the anticipated inter-study heterogeneity, a random-effects model was utilized to yield the summary statistics. Statistical heterogeneity was calculated using Cochran’s Q test and quantified by I^2^ statistic, with 25, 50, and 75% defining threshold for low, moderate and high heterogeneity, respectively. To explore the sources of heterogeneity and the potentially significant associations, subgroup analyses stratified by mean GA and BW, sample size, study design, administration regiment, country economic levels, and adjusted factors were conducted. Further, we performed a random-effects meta-regression in which the predefined covariates were available in more than 10 studies, including sample size, study design, sex, multiple pregnancy, need for mechanical ventilation, and surfactant, RDS, bronchopulmonary dysplasia (BPD), NEC, patent ductus arteriosus (PDA), IVH III/IV, sepsis, and mortality. A *p* value of < 0.05 (0.10 for Q test) was considered statistically significant.

To test the robustness of the results, we used sensitivity analysis by sequentially omitting one study at a time and combining the pooled ORs of the remainders. Publication bias was assessed through visual funnel plot inspection and quantified by Egger’s test, where a *p* value of < 0.05 indicated significant publication bias (Egger et al., 1997). If publication bias was observed, the “trim and fill” analysis would be performed to adjust the funnel plot asymmetry and recalculate the filled estimates.

## Results

### Search Results and Description of Studies

**FIGURE 1:**
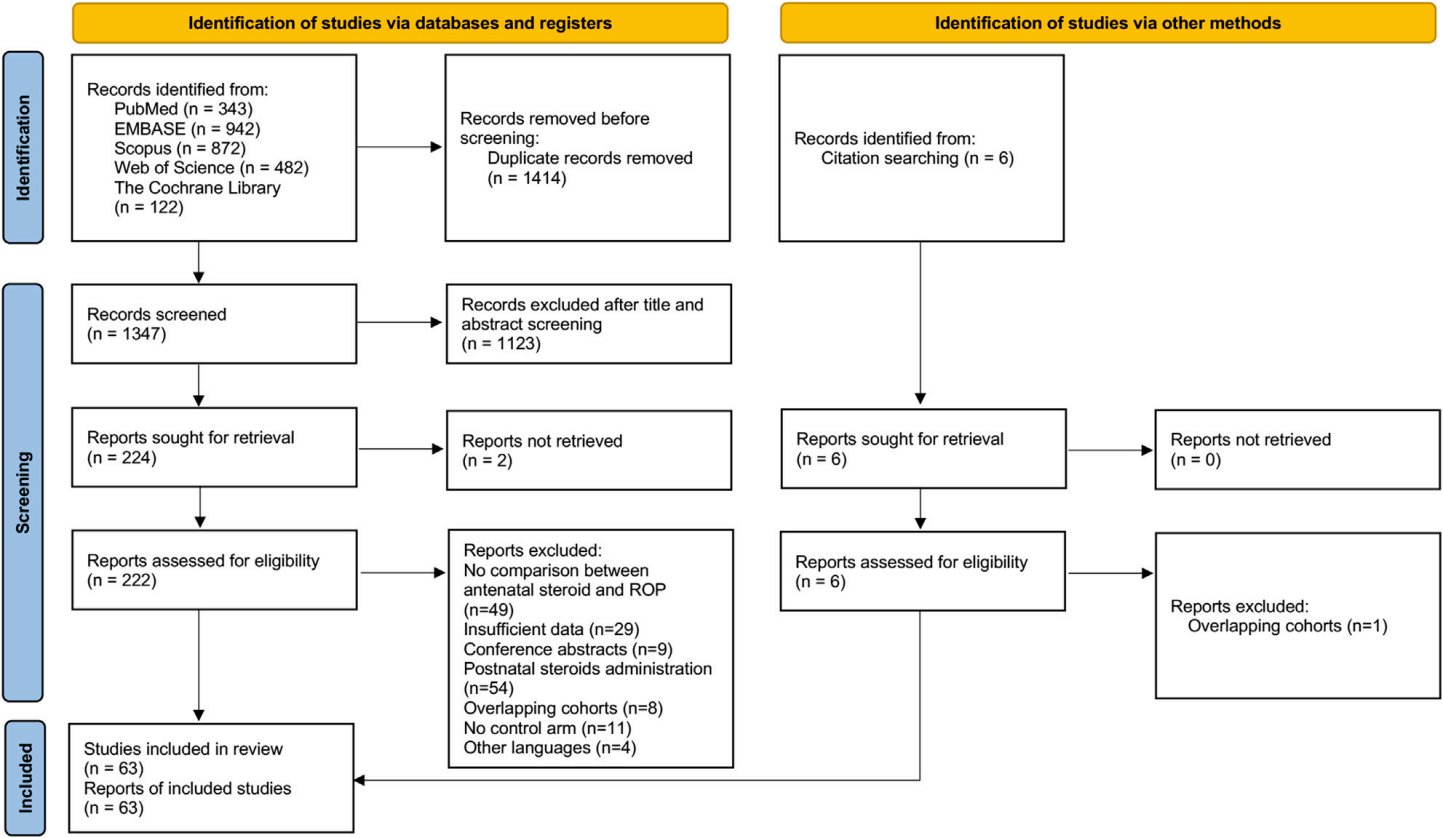
PRISMA flow diagram of the systematic search process.

Of 1,347 potentially relevant studies identified from the initial search after removing duplicates, 1,123 records were excluded after title and abstract screening. 222 full-text records were examined for eligibility and additional 5 records were identified from other sources (Dammann et al., 2009; Liu et al., 2012; Güran et al., 2013; Bas et al., 2018; Dani et al., 2021). Following detailed evaluation, 63 records were included for meta-analysis. The PRISMA flowchart of the database search was depicted in Figure 1 (Page et al., 2021).

The 63 included studies yielded 196,264 infants, with 18,260 cases of any ROP and 5,484 cases of severe ROP. The details of all included studies were summarized in Supplementary Table S2. No RCTs comparing ACS-treated with untreated neonates were identified from the search process. 40 studies were designed from the perspective of ROP, where ACS was one of the predetermined factors of ROP. 23 studies were designed from the perspective of ACS, where neonates with and without ACS were examined for neonatal morbidities including ROP. Of the 23 cohorts, 3 cohorts primarily reported the association between ACS and ROP (Console et al., 1997; Higgins et al., 1998; Maini et al., 2014). Mean GA and BW of all eligible studies ranged from 25 to 32.7 weeks and 696.7–1837.3 g, respectively. 24 studies reported effect sizes or numerical data based on the type of ACS (betamethasone or dexamethasone). Administration regiment of either complete or partial course was available in 25 studies. 38 studies looked at the severity of ROP. The classification and severity of ROP were mostly based on the International Classification for Retinopathy of Prematurity (ICROP) (Chiang et al., 2021).

### Risk of Bias Assessment

Since only observational studies were identified, we used Newcastle−Ottawa Scale (NOS) to assess the risk of bias. The analysis indicated that all included 63 studies attained a total NOS score of 5–9 (median = 7) (Supplementary Table S3).

### Antenatal Steroid Exposure and Any Stage ROP

**FIGURE 2:**
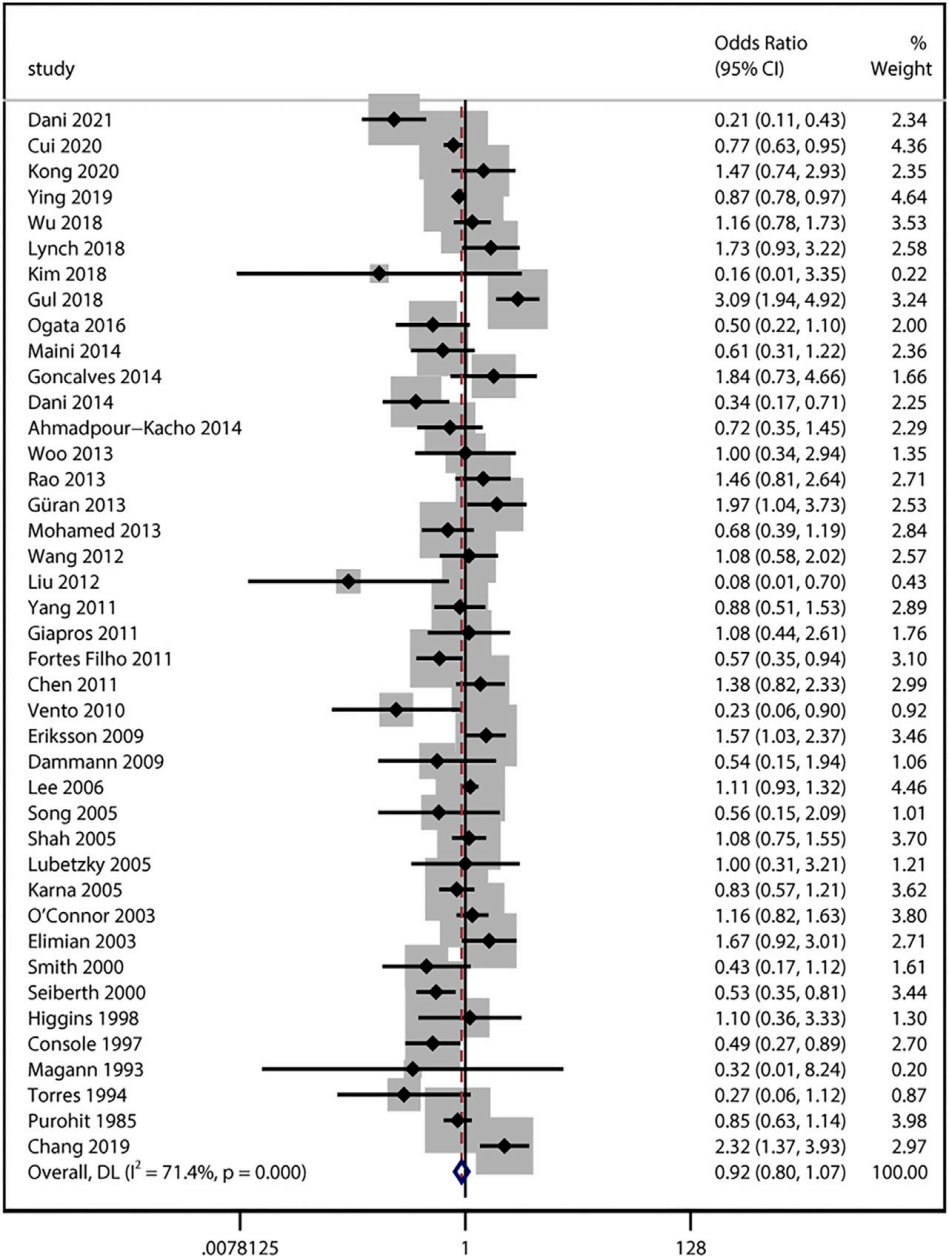
Meta-analysis of unadjusted ORs and 95% CIs for the association between antenatal corticosteroids exposure and any stage of retinopathy of prematurity.

40 studies provided numerical counts on ACS exposure between any stage of ROP and non-ROP groups. For the study by Chang (2019), only unadjusted OR based on univariate analysis was reported. The unadjusted ORs from the above 41 studies with a total of 34,302 infants were pooled. We found no significant association between ACS exposure and the any stage ROP development (unadjusted uOR 0.92, 95% CI 0.80–1.07, I^2^ = 71.4%, *p* = 0.29; Figure 2). Sensitivity analyses revealed no obvious changes of unadjusted odds after sequentially removing each study (I^2^ ranging from 66% to 72%, *p* value ranging from 0.11 to 0.57).

**FIGURE 3:**
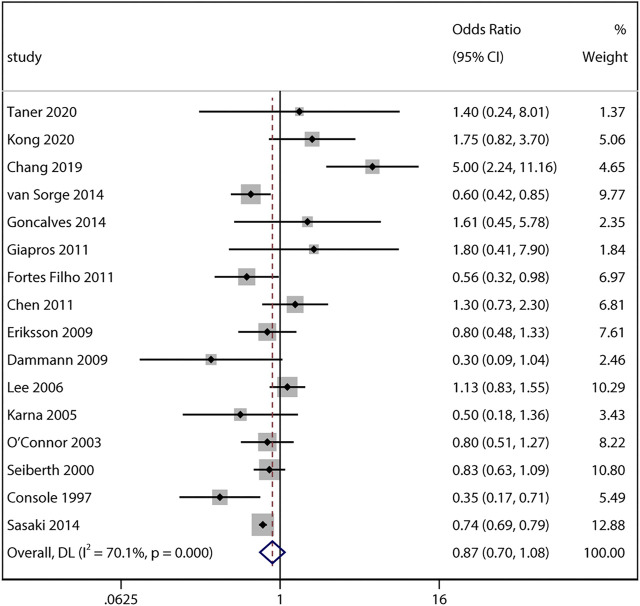
Meta-analysis of adjusted ORs and 95% CIs for the association between antenatal corticosteroids exposure and any stage of retinopathy of prematurity.

By pooling adjusted data from 16 studies, the result also yielded no significant difference between ACS exposure and non-ACS infants (adjusted aOR 0.87, 95% CI 0.7–1.08, I^2^ = 70.1%, *p* = 0.22; Figure 3). Sensitivity analyses revealed no obvious changes of adjusted odds after sequentially removing each study (I^2^ ranging from 52% to 72%, *p* value ranging from 0.12 to 0.49).

### Antenatal Steroid Exposure and Severe ROP

**FIGURE 4:**
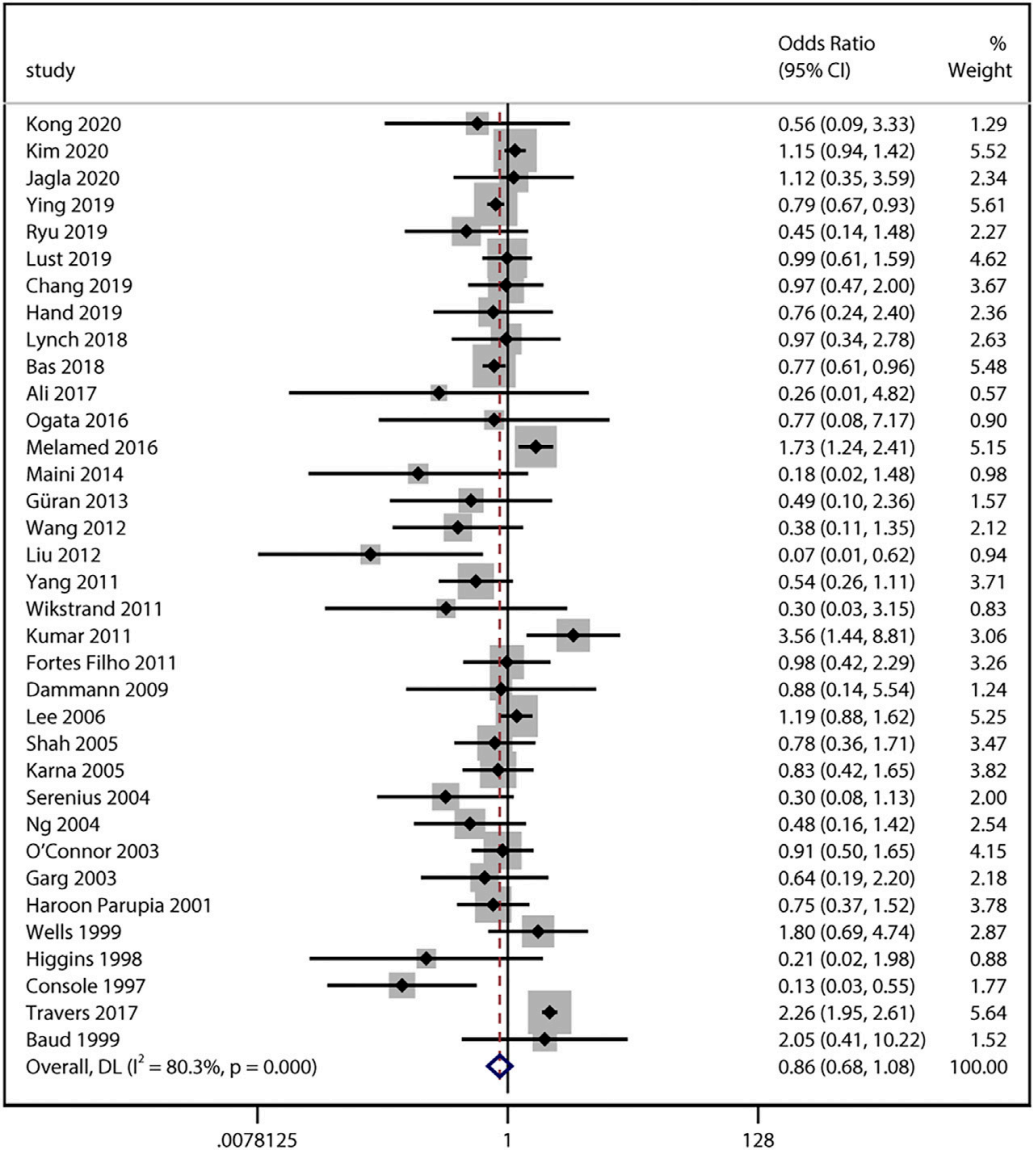
Meta-analysis of unadjusted ORs and 95% CIs for the association between antenatal corticosteroids exposure and severe retinopathy of prematurity.

33 studies reported numerical counts on the comparison between ACS exposure and severe ROP. Studies by Chang (2019) and Serenius et al. (2004) provided unadjusted ORs and their 95% CI instead. Of the above 35 studies with a total of 157,064 infants, 15 studies looked at infants who developed ROP requiring treatment (Wells et al., 1999; Shah et al., 2005; Lee et al., 2006; Fortes Filho et al., 2011; Kumar et al., 2011; Yang et al., 2011; Melamed et al., 2016; Ogata et al., 2016; Ali et al., 2017; Travers et al., 2017; Bas et al., 2018; Lust et al., 2019; Ryu et al., 2019; Ying et al., 2019; Kim et al., 2020). All the unadjusted data was pooled and the result also revealed no significant association between the two variables (uOR 0.86, 95% CI 0.68–1.08, I^2^ = 80.3%, *p* = 0.19; Figure 4). Sensitivity analysis was conducted and no obvious changes of pooled odds were observed (I^2^ ranging from 51 to 81%, *p* value ranging from 0.10 to 0.32).

**FIGURE 5:**
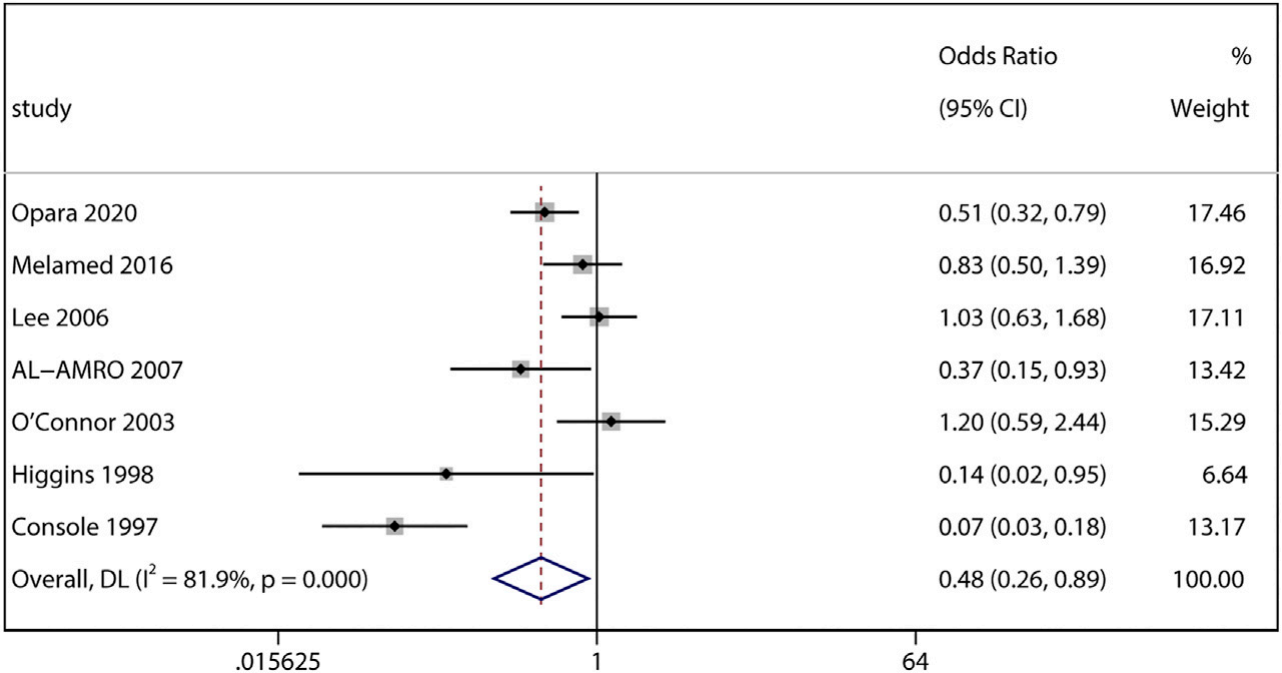
Meta-analysis of adjusted ORs and 95% CIs for the association between antenatal corticosteroids exposure and severe retinopathy of prematurity.

Adjusted data provided from 7 studies was subsequently pooled. Interestingly, we found the adjusted odds of severe ROP (aOR 0.48, 95% CI 0.26–0.89, I^2^ = 81.9%, *p* = 0.02; Figure 5) were significantly lower in ACS infants compared with non-ACS infants. Sensitivity analysis revealed the association became marginally insignificant after excluding Opara et al. (aOR 0.46, 95% CI 0.21–1.01, I^2^ = 84.3%, *p* = 0.05) and Console et al. (aOR 0.69, 95% CI 0.46–1.05, I^2^ = 56.9%, *p* = 0.08) individually.

### Subgroup Analyses

Subgroup analysis based on unadjusted data was conducted to unveil the potential influence of other study characteristics. Results from prespecified subgroups were shown in Table 1. Significantly lower risks of any stage ROP were found in extremely preterm infants (EPI, GA < 28 weeks) (uOR 0.65, 95% CI 0.44–0.95, I^2^ = 74.7%, *p* = 0.03) and extremely low birth weight infants (ELBW, BW < 1,000 g) (uOR 0.60, 95% CI 0.38–0.93, I^2^ = 77.8%, *p* = 0.02). Moreover, a significant association between the two variables was observed in studies with sample sizes less than 500 (uOR 0.73, 95% CI 0.56–0.96, I^2^ = 67.9%, *p* = 0.02). No statistical difference was detected when stratified by other factors. Subgroup analysis for studies adjusted for RDS or oxygen therapy was also performed for any stage ROP. Pooled results of eight included studies remained insignificant (aOR 0.97, 95% CI 0.55–1.72, I^2^ = 76.9%, *p* = 0.93).

**TABLE 1:** Subgroup analysis of any stage ROP risk in the ACS-exposed group.

ROP type	Subgroup	Number of studies	OR (95%CI)	*p* value	I^2^	P for subgroup difference
Any ROP	Mean GA					
	<28 weeks	12	0.65 [0.44, 0.95]	**0.03**	74.7	0.03
	≥28 weeks	26	1.04 [0.87, 1.24]	0.70	70.5	
	Mean BW					
	≤1,000 g	9	0.60 [0.38, 0.93]	**0.02**	77.8	0.03
	>1,000 g	28	1.01 [0.85, 1.21]	0.88	70	
	Study design					
	Case-control	27	0.91 [0.75, 1.10]	0.31	74.3	0.74
	Cohort	14	0.96 [0.73, 1.25]	0.75	64.7	
	Drug type					
	Dexamethasone	9	0.90 [0.73, 1.11]	0.31	25.3	0.67
	Betamethasone	12	0.84 [0.65, 1.07]	0.16	57.4	
	ACS course					
	Complete	16	0.85 [0.71, 1.02]	0.08	58.9	0.21
	Partial	4	0.99 [0.85, 1.14]	0.85	12.6	
	Sample size					
	<500	27	0.73 [0.56, 0.96]	**0.02**	67.9	0.01
	≥500	14	1.11 [0.94, 1.31]	0.21	74.8	
	Economic Level					
	Developing	20	1.04 [0.83, 1.30]	0.73	69.8	0.13
	Developed	21	0.82 [0.66, 1.01]	0.07	71.6	
	Adjusted for RDS/oxygen					
	YES	8	0.97 [0.55, 1.72]	0.93	76.9	0.71
	NO	7	0.87 [0.67, 1.12]	0.27	68.6	

OR, odds ratio; CI, confidence interval; ROP, retinopathy of prematurity; ACS, antenatal corticosteroids; GA, gestational age; BW, birth weight; RDS, respiratory distress syndrome.

The bold values indicate statistically significant results.

As for the association between ACS and severe ROP, significantly lower risks were found in studies with sample sizes smaller than 500 (uOR 0.63, 95% CI 0.50–0.80, *p* < 0.0001), and the heterogeneity reduced to 0%. We also noted a significant association in case-control studies (uOR 0.79, 95% CI 0.66–0.94, *p* = 0.01) with reduced heterogeneity to 26.9%. However, no significant between-subgroup deference was detected (*p* = 0.06). There were no special positive findings of other aforementioned factors (Table 2). Three studies were included for subgroup analysis adjusted for RDS or oxygen therapy, and the pooled adjusted results remained significant (aOR 0.18, 95% CI 0.04–0.82, I^2^ = 86%, *p* = 0.03).

**TABLE 2:** Subgroup analysis of severe ROP risk in the ACS-exposed group.

ROP type	Subgroup	Number of studies	OR (95%CI)	*p* value	I^2^	P for subgroup difference
Severe ROP	Mean GA					
	<28 weeks	6	0.67 [0.40, 1.10]	0.11	30	0.22
	≥28 weeks	12	0.97 [0.70, 1.34]	0.86	62.1	
	Mean BW					
	≤1,000 g	5	0.82 [0.55, 1.22]	0.32	0	0.65
	>1,000 g	13	0.92 [0.69, 1.22]	0.56	69.8	
	Study design					
	Case-control	23	0.79 [0.66, 0.94]	**0.01**	26.9	0.06
	Cohort	12	1.14 [0.81, 1.62]	0.44	79.5	
	Drug type					
	Dexamethasone	6	0.81 [0.46, 1.41]	0.45	26.5	0.66
	Betamethasone	8	0.94 [0.66, 1.32]	0.70	26.2	
	ACS course					
	Complete	11	0.93 [0.66, 1.32]	0.70	70.1	0.52
	Partial	4	1.15 [0.67, 2.00]	0.61	44.5	
	Sample size					
	<500	22	0.63 [0.50, 0.80]	**<0.0001**	0	0.00
	≥500	12	1.28 [0.93, 1.78]	0.14	91	
	Economic Level					
	Developing	18	0.89 [0.62, 1.28]	0.54	86	0.91
	Developed	18	0.87 [0.72, 1.05]	0.16	22	
	Adjusted for RDS/oxygen					
	YES	3	0.18 [0.04, 0.82]	**0.03**	86	0.05
	NO	4	0.86 [0.58, 1.27]	0.44	35	

OR, odds ratio; CI, confidence interval; ROP, retinopathy of prematurity; ACS, antenatal corticosteroids; GA, gestational age; BW, birth weight; RDS, respiratory distress syndrome.

The bold values indicate statistically significant results.

### Meta-Regression Analyses

**FIGURE 6:**
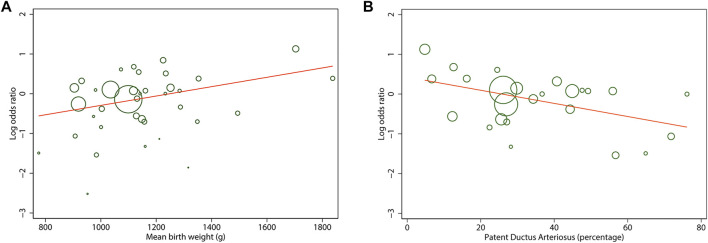
Meta-regression plot of mean birth weight **(A)** and patent ductus arteriosus rate **(B)** of the cohort on the log odds ratio in any stage of retinopathy of prematurity between antenatal corticosteroids exposed and non-exposed infants. Each circle size is proportional to the study weight.

To address the potential sources of heterogeneity, random-effects meta-regression was conducted for analyses involving more than 10 studies. Results of all univariate analysis are shown in Supplementary Table S4. For any stage ROP, we found that mean BW of the cohort significantly explained 20% of variance across studies (*p* = 0.02). Each increment of 100 g in the BW of the cohort resulted in an increase in any stage ROP log OR of 0.11 (Figure 6A). Similarly, the rate of PDA of the cohort significantly explained 31% of the heterogeneity (*p* = 0.02), indicating each 10% decrease in PDA rate across studies associated with an increase in any stage ROP log OR of 0.2 (Figure 6B). However, multivariate meta-regression analysis based on data from all studies evaluating those two covariates (*k* = 25) revealed non-significant result (*p* = 0.08 for mean BW, *p* = 0.18 for PDA rate).

For severe ROP, sample size of the cohort significantly explained 58% (*p* = 0.003) of variance. One study, with a relatively large sample size (*n* = 117,941), played a dominate role in this effect (Travers et al., 2017). Thus, additional analysis without the study was conducted and the result remained significant (*R* ^2^ = 46%, *p* = 0.02). Study design of the cohort also explained a significant proportion of variance (*R* ^2^ = 49%, *p* = 0.03). Multivariate meta-regression including those two covariates (*k* = 35) failed, probably due to the same large study as an outlier. Additional multivariate analysis without this study showed non-significant result (*k* = 34, *p* = 0.12 for sample size, *p* = 0.30 for study design), indicating their associations with the effect size were interdependent.

### Publication Bias

**FIGURE 7:**
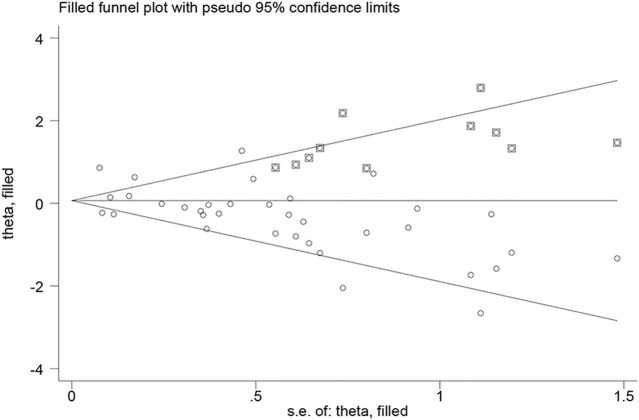
Filled funnel plot of unadjusted data for severe ROP showing number of potential missing studies.

Neither visual inspection of funnel plots nor Egger’s test suggested publication bias (*p* > 0.05) except for pooled unadjusted data for severe ROP (Egger’s test, *p* = 0.01) (Supplementary Figure S1). Therefore, we performed “trim and fill” analysis to estimate the missing studies. 11 potential missing studies were identified by adjusting funnel plot asymmetry (Figure 7). Filled estimates remained insignificant as well (filled uOR 1.06, 95% CI 0.85–1.34, *p* = 0.57).

## Discussion

Our present study is a substantial update of the previous meta-analysis by Yim et al. (2018), with a greater pool of studies (63 vs. 28), a much larger number of infants (196,264 vs. 20,731), and a more comprehensive analysis of covariates. For the current meta-analysis, the substantial increase in the number of included studies largely stems from its up-to-date nature, along with a wider range of databases and a modified search strategy. Five large-scale studies (*n* > 5,000) were also identified since 2014. Owing to the lack of available RCTs, our meta-analysis of observational studies provides the current best evidence on the association of ACS exposure and ROP. After integrating all available studies, we demonstrated no significant association between ACS exposure and any stage ROP. Results were more variable concerning severe ROP. Unadjusted analysis showed similar nonsignificant effects, while adjusted data revealed that ACS-exposure infants were at significantly reduced risks of severe ROP development.

One important finding in our subgroup analysis was that ACS at extreme preterm gestations could prevent the occurrence of ROP, rather than ROP progression. Various efforts have been made to prevent ROP in this particularly high-risk birth cohort (Bulka et al., 2019; Mayock et al., 2020). Unlike other morbidities of prematurity, the magnitude of benefits of ACS on ROP for immature infants is confusing, in part due to limited number of eligible infants and variable confounders (Melamed et al., 2015; Chawla et al., 2016). A large Chinese cohort reported that ACS treatment significantly decreased ROP risks for 1,544 immature infants. However, the association was no longer significant when EPI and ELBW infants were separately analyzed (Cui, 2020). O’Connor et al. reported ACS as a risk factor of ROP in unadjusted data for infants <1,250 g weight (uOR 1.38), which switched to a protective factor in adjusted analysis (aOR 0.81) (O’Connor et al., 2003). Our subgroup analyses integrating ORs from studies with a total of 4,326 EPI and 3,547 ELBW infants provided much stronger evidence. As stressed in a systemic review, we suggest routine usage of ACS in pregnancies at extremely preterm gestations to prevent neonatal morbidities of prematurity, including ROP (Jobe and Goldenberg, 2018). A caution is that the baseline ROP risk for infants at higher gestations is already minimized, thus, the statistical power of the present study might fail to detect the subtle difference.

Unlike mild ROP, infants with severe ROP are prone to develop irreversible vision loss (Cayabyab and Ramanathan, 2016). Growing evidence has suggested ACS may decrease, but not prevent, the severity of ROP (Console et al., 1997; Bas et al., 2018; Chang, 2019). In our unadjusted analysis, we observed no protective effect of ACS on ROP progression. Although the association becomes significant in case-control studies, no between-subgroup difference was detected. Hence, one can speculate that the higher weight of case-control studies in the meta-analysis may lead to a false-positive conclusion. It is generally believed that case-control studies have higher risk of bias, for the inability to control for important confounding factors (i.e., prematurity) (Omair, 2016). Furthermore, the population of case-control studies is much smaller than that of cohort studies (15,064 vs. 142,048), thereby evidence simply from case-control studies may be less convincing. Contrary to unadjusted data, significant association was identified in the adjusted analysis. The result should be interpreted with caution in consideration of limited studies with substantial heterogeneity. Taken together, although there seems to be an association between ACS and the severity of ROP based on our adjusted analysis, more evidence adjusted for common confounders are required to draw a definite conclusion.

We performed further hypothesis-generating analyses considering possible benefits from varying exposure of ACS regimens. Although the therapy is extremely variable in low-income countries, no significant differences in ROP outcomes were detected based on economic levels (Vogel et al., 2014). We focused further on the differences between studies in drug doses and types. As preterm labor may occur prior to completing a course, partial dosing of ACS is expected especially in studies with no details of regimens (Chawla et al., 2010). Chances are studies administrating partial courses are associated with no or less improvement in ROP, masking the potential benefits of complete courses in meta-analysis (Salhab et al., 2003; Ying et al., 2019). Although subgroup analysis failed to reveal a significant association in complete and partial groups, the effect sizes exhibited a dose-dependent manner for ROP development as in other morbidities (Chawla et al., 2016). Similar insignificant results also existed in both betamethasone and dexamethasone groups, which to some extent, due to the fact that they are epimers thus function equally for women at risk of preterm labor (American College of Obstetricians and Gynecologists’ Committee on Practice Bulletins—Obstetrics, 2016; Booker and Gyamfi-Bannerman, 2018). We did not look at repeated courses of ACS as they were only evaluated in 2 studies, showing no significant protective effects on ROP (Smith et al., 2000; Karna et al., 2005).

Considerable differences in ACS effect on ROP incidence were present across studies. Thus, meta-regression analyses were performed to evaluate how these confounders, such as prematurity, contributed to the overall heterogeneity. Univariate meta-regression showed mean BW and PDA rate of the cohort significantly modified the effect of ACS on any ROP development. That is, those studies including infants with lower BW and higher PDA rates might result in a stronger protective effect of ACS on the occurrence of ROP. Although the two covariates changed to nonsignificant in multivariate meta-regression, the results are less reliable with only 25 studies available. We can speculate, at least in part, that the potential protective effect of ACS on ROP can be masked by the diverse subject characteristics. Proper control of these two covariates is thereby essential for the future studies. Besides, the association between ACS and severe ROP was stronger in case-control studies with smaller sample size, corresponding to the subgroup analysis. In that case, the overweight of those study designs in the meta-analysis may produce false-positive results (Hackshaw, 2008). Those confounders may partly explain the significant positive results derived from Yim et al. (2018). As early RCTs have demonstrated significant improvements of neonatal outcomes for impending premature labor, performing RCTs on this subject would therefore be unethical (Roberts et al., 2017). Large-scale cohort studies are thereby needed to establish credibility of future analysis.

The protective effect of ACS on ROP is controversial yet biologically plausible. ACS have long been suggested to modulate fetal maturity, thus may accelerate maturation of retinal vasculature as well (Higgins et al., 1998; Al-Amro et al., 2007). However, such mechanism fails to explain their null effects on ROP occurrence observed in the present study. In literature, whether ACS acts directly on the risk of ROP remains controversial (Console et al., 1997; Chang, 2019). A plausible explanation could be the fact that ACS reduces the risk of RDS and thus the demand for supplemental oxygen. It appears to be convincing because RDS and oxygen therapy have long been recognized as risk factors for any and severe ROP (Chen et al., 2010; Alajbegovic-Halimic et al., 2015; Kim et al., 2018). Nevertheless, our subgroup analysis for three studies adjusted for RDS and/or oxygen therapy also revealed a significant protective role of ACS on severe ROP. By conducting similar analysis, Yim et al. likewise suggested their positive results of ACS on any ROP might be independent of RDS/oxygen status (Yim et al., 2018). In that case, the role of RDS and oxygen therapy in the association between ACS and ROP might not be as important as they seem. However, the limited data on this issue requires more comparative studies adjusted for these confounders to reach a more reliable conclusion. We postulate another possible mechanism for the protective effect of ACS on severe ROP. ACS are also potent suppressors of inflammatory responses in the newborn during the first days after birth (Kemp et al., 2016). Following ACS treatment, immune cells in babies, especially neutrophils and macrophages, tend to demarginate from vascular wall and transiently reduce the production of cytokines (i.e., interlutin-6 (IL-6), IL-10 and tumor necrosis factor alpha (TNF-α)), chemokines and reactive oxygen species (ROS) (Fay et al., 2016; Holm et al., 2017; Perna-Barrull et al., 2020). Animal studies have demonstrated those molecules, especially IL-6 and TNF-α, are involved in sensitizing the developing retinal blood vessels to injuries such as oxygen changes, and cause subsequent neovascularization (Tremblay et al., 2013; Hong et al., 2014). Clinical evidence from protein profiling in the eye and cord blood serum also identified them as potential biomarkers for ROP progression, as they are reversely associated with IGF-1 levels (Hellgren et al., 2018; Lyu et al., 2018; Park et al., 2019). Rathi et al. further detected complement activation in the vitreous of proliferative ROP patients (Rathi et al., 2017). Of note, whether ACS bind to GR in circulating immune cells and cross the destructed blood-retinal barrier, or directly bind to retinal immune cells (i.e., microglia) and reduce cytokines secretion requires further investigations. Moreover, studies also attest to the capacity of ACS to reduce T cell proliferation in the newborn, thereby may ameliorate the aberrant increase of T lymphocytes during the neovascularization stage of ROP (Kavelaars et al., 1999; Talia et al., 2013; Deliyanti et al., 2017). Taken together, one can speculate an immune-vascular interaction is predominantly present in the pathway from ACS to more advanced stage of ROP. That is, ACS may directly impact the inflammation pathway, and subsequently modify the vasculature profile in newborns at risk of severe ROP.

The present meta-analysis has several strengths. First, this is the largest meta-analysis to date examining the risk of ROP in ACS-exposed infants. We include 63 cohort studies involving infants at gestational week from 25 to 32.7 weeks. Results are therefore applicable to the general populations of preterm infants born to mothers with standard care of ACS (Committee on Obstetric Practice, 2017). Second, we well-integrate data from the subgroup of extremely immature infants and provide stronger evidence on this particular high-risk population. Lastly, extensive confounding factors, especially varying exposure of ACS regimens, are taken into consideration so as to better explain the heterogeneity and interpret our findings.

Certain limitations of the present study should be considered. First, the results are based on observational studies because RCTs are not available on this issue. One should be notified the results from such study design do not provide true assessment of causality between the two variables, as it is subjected to potential bias and confounding. For example, we acknowledge that there might be risk of selection bias if mothers who are potential candidates for ACS are not treated with steroids, because of maternal or fetal emergencies. Those non-treated infants might suffer circulatory or/and respiratory instability, thereby at greater risk of developing ROP (Lee and Dammann, 2012). However, it is impossible to identify those bias as relevant data reporting emergency delivery is only available in one study (Wang et al., 2012). To mitigate the inherent handicap of such design in terms of risk of bias, more studies adjusting for major confounders are thus warranted. Second, missing data for covariates leading to a source of bias is another issue of concern, especially for ACS regimens and certain morbidities. Although meta-regression results are only applicable for a specific subsample, the number of available studies for most covariates is yet large enough to detect the between-study heterogeneity. Third, publication bias is expected for unadjusted data of severe ROP. Of note, 5 relevant studies were retrieved by hand search, which implies additional studies might be missed by our search strategy possibly because severe ROP is always a secondary outcome in most studies. Nevertheless, the “trim and fill” analysis filling 11 missing studies attests to the robustness of our results.

## Conclusion

The current meta-analysis based on robust evidence confirmed that ACS could not prevent the occurrence of ROP in the general population, but are protective in extremely immature infants. We thereby suggest routine usage of ACS in expected extremely premature infants to prevent ROP occurrence. We further observed that ACS might decrease the severity of ROP, which still warrants further investigations to reach a more reliable conclusion.

## Data Availability

The raw data supporting the conclusions of this article will be made available by the authors, without undue reservation.

## References

[B1] Alajbegovic-Halimic J. Zvizdic D. Alimanovic-Halilovic E. Dodik I. Duvnjak S. (2015). Risk Factors for Retinopathy of Prematurity in Premature Born Children. Med. Arch. 69 (6), 409–413. 10.5455/medarh.2015.69.409-413 26843736 PMC4720470

[B2] Al-Amro S. A. Al-Kharfi T. M. Thabit A. A. Al-Mofada S. M. (2007). Risk Factors for Acute Retinopathy of Prematurity. Compr. Ther. 33 (2), 73–77. 10.1007/s12019-007-8008-5 18004017

[B3] Ali A. A. Gomaa N. A. S. Awadein A. R. Al-Hayouti H. H. Hegazy A. I. (2017). Retrospective Cohort Study Shows that the Risks for Retinopathy of Prematurity Included Birth Age and Weight, Medical Conditions and Treatment. Acta Paediatr. 106 (12), 1919–1927. 10.1111/apa.14019 28799178

[B4] American College of Obstetricians and Gynecologists’ Committee on Practice Bulletins—Obstetrics (2016). Practice Bulletin No. 171: Management of Preterm Labor. Obstet. Gynecol. 128 (4)**,** e155–64. 10.1097/aog.0000000000001711 27661654

[B5] Bas A. Y. Demirel N. Koc E. Ulubas Isik D. Hirfanoglu İ. M. Tunc T. (2018). Incidence, Risk Factors and Severity of Retinopathy of Prematurity in Turkey (TR-ROP Study): A Prospective, Multicentre Study in 69 Neonatal Intensive Care Units. Br. J. Ophthalmol. 102 (12), 1711–1716. 10.1136/bjophthalmol-2017-311789 29519879 PMC6287567

[B6] Blencowe H. Lawn J. E. Vazquez T. Fielder A. Gilbert C. (2013). Preterm-Associated Visual Impairment and Estimates of Retinopathy of Prematurity at Regional and Global Levels for 2010. Pediatr. Res. 74 Suppl 1 (Suppl. 1), 35–49. 10.1038/pr.2013.205 24366462 PMC3873709

[B7] Booker W. A. Gyamfi-Bannerman C. (2018). Antenatal Corticosteroids: Who Should We Be Treating? Clin. Perinatol 45 (2), 181–198. 10.1016/j.clp.2018.01.002 29747882

[B8] Bulka C. M. Dammann O. Santos H. P. Jr. VanderVeen D. K. Smeester L. Fichorova R. (2019). Placental CpG Methylation of Inflammation, Angiogenic, and Neurotrophic Genes and Retinopathy of Prematurity. Invest. Ophthalmol. Vis. Sci. 60 (8), 2888–2894. 10.1167/iovs.18-26466 31266060 PMC6607927

[B9] Cayabyab R. Ramanathan R. (2016). Retinopathy of Prematurity: Therapeutic Strategies Based on Pathophysiology. Neonatology 109 (4), 369–376. 10.1159/000444901 27251645

[B10] Chang J. W. (2019). Risk Factor Analysis for the Development and Progression of Retinopathy of Prematurity. PLoS One 14 (7), e0219934. 10.1371/journal.pone.0219934 31318921 PMC6638955

[B11] Chawla S. Natarajan G. Rane S. Thomas R. Cortez J. Lua J. (2010). Outcomes of Extremely Low Birth Weight Infants with Varying Doses and Intervals of Antenatal Steroid Exposure. J. Perinat Med. 38 (4), 419–423. 10.1515/jpm.2010.060 20297898

[B12] Chawla S. Natarajan G. Shankaran S. Pappas A. Stoll B. J. Carlo W. A. (2016). Association of Neurodevelopmental Outcomes and Neonatal Morbidities of Extremely Premature Infants with Differential Exposure to Antenatal Steroids. JAMA Pediatr. 170 (12), 1164–1172. 10.1001/jamapediatrics.2016.1936 27723868 PMC5294968

[B13] Chen M. L. Guo L. Smith L. E. Dammann C. E. Dammann O. (2010). High or Low Oxygen Saturation and Severe Retinopathy of Prematurity: a Meta-Analysis. Pediatrics 125 (6), e1483–92. 10.1542/peds.2009-2218 20498174 PMC4016714

[B14] Chiang M. F. Quinn G. E. Fielder A. R. Ostmo S. R. Paul Chan R. V. Berrocal A. (2021). International Classification of Retinopathy of Prematurity, Third Edition. Ophthalmology 128, e51–e68. 10.1016/j.ophtha.2021.05.031 34247850 PMC10979521

[B15] Committee on Obstetric Practice (2017). Committee Opinion No. 713 Summary: Antenatal Corticosteroid Therapy for Fetal Maturation. Obstet. Gynecol. 130 (2), 493–494. 10.1097/aog.0000000000002231 28742672

[B16] Console V. Gagliardi L. De Giorgi A. De Ponti E. (1997). Retinopathy of Prematurity and Antenatal Corticosteroids. The Italian ROP Study Group. Acta Biomed. Ateneo Parmense 68 Suppl 1 (Suppl. 1), 75–79. 10021720

[B17] Cui Q. (2020). Antenatal Corticosteroid Administration in Extremely Preterm and Extremely Low Birth Weight Infants and its Effects on Prognosis: A Multicentre Survey. Chin. J. Perinat Med. 23 (5), 302–310. 10.3760/cma.j.cn113903-20190823-00512

[B18] Dammann O. Brinkhaus M. J. Bartels D. B. Dördelmann M. Dressler F. Kerk J. (2009). Immaturity, Perinatal Inflammation, and Retinopathy of Prematurity: a Multi-Hit Hypothesis. Early Hum. Dev. 85 (5), 325–329. 10.1016/j.earlhumdev.2008.12.010 19217727

[B19] Dani C. Coviello C. Panin F. Frosini S. Costa S. Purcaro V. (2021). Incidence and Risk Factors of Retinopathy of Prematurity in an Italian Cohort of Preterm Infants. Ital. J. Pediatr. 47 (1), 64. 10.1186/s13052-021-01011-w 33712037 PMC7953747

[B20] Deliyanti D. Talia D. M. Zhu T. Maxwell M. J. Agrotis A. Jerome J. R. (2017). Foxp3+ Tregs Are Recruited to the Retina to Repair Pathological Angiogenesis. Nat. Commun. 8 (1), 748. 10.1038/s41467-017-00751-w 28963474 PMC5622066

[B21] Early Treatment For Retinopathy Of Prematurity Cooperative Group (2003). Revised Indications for the Treatment of Retinopathy of Prematurity: Results of the Early Treatment for Retinopathy of Prematurity Randomized Trial. Arch. Ophthalmol. 121 (12), 1684–1694. 10.1001/archopht.121.12.1684 14662586

[B22] Egger M. Davey Smith G. Schneider M. Minder C. (1997). Bias in Meta-Analysis Detected by a Simple, Graphical Test. Bmj 315 (7109), 629–634. 10.1136/bmj.315.7109.629 9310563 PMC2127453

[B23] Eriksson L. Haglund B. Ewald U. Odlind V. Kieler H. (2009). Short and Long-Term Effects of Antenatal Corticosteroids Assessed in a Cohort of 7,827 Children Born Preterm. Acta Obstet. Gynecol. Scand. 88 (8), 933–938. 10.1080/00016340903111542 19568962

[B24] Fay M. E. Myers D. R. Kumar A. Turbyfield C. T. Byler R. Crawford K. (2016). Cellular Softening Mediates Leukocyte Demargination and Trafficking, Thereby Increasing Clinical Blood Counts. Proc. Natl. Acad. Sci. U S A. 113 (8), 1987–1992. 10.1073/pnas.1508920113 26858400 PMC4776450

[B25] Fortes Filho J. B. Costa M. C. Eckert G. U. Santos P. G. Silveira R. C. Procianoy R. S. (2011). Maternal Preeclampsia Protects Preterm Infants against Severe Retinopathy of Prematurity. J. Pediatr. 158 (3), 372–376. 10.1016/j.jpeds.2010.08.051 20888573

[B26] Güran Ö. Bülbül A. Uslu S. Dursun M. Zubarioğlu U. Nuhoğlu A. (2013). The Change of Morbidity and Mortality Rates in Very Low Birth Weight Infants over Time. Turk Pediatri Arsivi 48, 102–109. 10.4274/tpa.1062

[B27] Hackshaw A. (2008). Small Studies: Strengths and Limitations. Eur. Respir. J. 32 (5), 1141–1143. 10.1183/09031936.00136408 18978131

[B28] Hamling J. Lee P. Weitkunat R. Ambühl M. (2008). Facilitating Meta-Analyses by Deriving Relative Effect and Precision Estimates for Alternative Comparisons from a Set of Estimates Presented by Exposure Level or Disease Category. Stat. Med. 27 (7), 954–970. 10.1002/sim.3013 17676579

[B29] Hartnett M. E. (2017). Advances in Understanding and Management of Retinopathy of Prematurity. Surv. Ophthalmol. 62 (3), 257–276. 10.1016/j.survophthal.2016.12.004 28012875 PMC5401801

[B30] Hellgren G. Löfqvist C. Hansen-Pupp I. Gram M. Smith L. E. Ley D. (2018). Increased Postnatal Concentrations of Pro-inflammatory Cytokines Are Associated with Reduced IGF-I Levels and Retinopathy of Prematurity. Growth Horm. IGF Res. 39, 19–24. 10.1016/j.ghir.2017.11.006 29274846 PMC5858996

[B31] Higgins R. D. Mendelsohn A. L. DeFeo M. J. Ucsel R. Hendricks-Munoz K. D. (1998). Antenatal Dexamethasone and Decreased Severity of Retinopathy of Prematurity. Arch. Ophthalmol. 116 (5), 601–605. 10.1001/archopht.116.5.601 9596495

[B32] Holm M. Morken T. S. Fichorova R. N. VanderVeen D. K. Allred E. N. Dammann O. (2017). Systemic Inflammation-Associated Proteins and Retinopathy of Prematurity in Infants Born before the 28th Week of Gestation. Invest. Ophthalmol. Vis. Sci. 58 (14), 6419–6428. 10.1167/iovs.17-21931 29260199 PMC5736326

[B33] Hong H. K. Lee H. J. Ko J. H. Park J. H. Park J. Y. Choi C. W. (2014). Neonatal Systemic Inflammation in Rats Alters Retinal Vessel Development and Simulates Pathologic Features of Retinopathy of Prematurity. J. Neuroinflamm. 11, 87. 10.1186/1742-2094-11-87 PMC4030274 24886524

[B34] Jobe A. H. Goldenberg R. L. (2018). Antenatal Corticosteroids: An Assessment of Anticipated Benefits and Potential Risks. Am. J. Obstet. Gynecol. 219 (1), 62–74. 10.1016/j.ajog.2018.04.007 29630886

[B35] Karna P. Muttineni J. Angell L. Karmaus W. (2005). Retinopathy of Prematurity and Risk Factors: A Prospective Cohort Study. BMC Pediatr. 5 (1), 18. 10.1186/1471-2431-5-18 15985170 PMC1175091

[B36] Kavelaars A. van der Pompe G. Bakker J. M. van Hasselt P. M. Cats B. Visser G. H. (1999). Altered Immune Function in Human Newborns after Prenatal Administration of Betamethasone: Enhanced Natural Killer Cell Activity and Decreased T Cell Proliferation in Cord Blood. Pediatr. Res. 45 (3), 306–312. 10.1203/00006450-199903000-00003 10088646

[B37] Kemp M. W. Newnham J. P. Challis J. G. Jobe A. H. Stock S. J. (2016). The Clinical Use of Corticosteroids in Pregnancy. Hum. Reprod. Update 22 (2), 240–259. 10.1093/humupd/dmv047 26590298

[B38] Kim S. J. Port A. D. Swan R. Campbell J. P. Chan R. V. P. Chiang M. F. (2018). Retinopathy of Prematurity: A Review of Risk Factors and Their Clinical Significance. Surv. Ophthalmol. 63 (5), 618–637. 10.1016/j.survophthal.2018.04.002 29679617 PMC6089661

[B39] Kim J. K. Hwang J. H. Lee M. H. Chang Y. S. Park W. S. (2020). Mortality Rate-dependent Variations in Antenatal Corticosteroid-Associated Outcomes in Very Low Birth Weight Infants with 23-34 Weeks of Gestation: A Nationwide Cohort Study. PLoS One 15 (10), e0240168. 10.1371/journal.pone.0240168 33017428 PMC7535030

[B40] Kumar P. Sankar M. J. Deorari A. Azad R. Chandra P. Agarwal R. (2011). Risk Factors for Severe Retinopathy of Prematurity in Preterm Low Birth Weight Neonates. Indian J. Pediatr. 78 (7), 812–816. 10.1007/s12098-011-0363-7 21340729

[B41] Lee J. Dammann O. (2012). Perinatal Infection, Inflammation, and Retinopathy of Prematurity. Semin. Fetal Neonatal. Med. 17 (1), 26–29. 10.1016/j.siny.2011.08.007 21903492 PMC3242877

[B42] Lee B. H. Stoll B. J. McDonald S. A. Higgins R. D. (2006). Adverse Neonatal Outcomes Associated with Antenatal Dexamethasone versus Antenatal Betamethasone. Pediatrics 117 (5), 1503–1510. 10.1542/peds.2005-1749 16651303

[B43] Liggins G. C. Howie R. N. (1972). A Controlled Trial of Antepartum Glucocorticoid Treatment for Prevention of the Respiratory Distress Syndrome in Premature Infants. Pediatrics 50 (4), 515–525. 10.1542/peds.50.4.515 4561295

[B44] Liu Y.-S. Chen T.-C. Yang C.-H. Yang C.-M. Huang J.-S. Ho T.-C. (2012). Incidence, Risk Factors, and Treatment of Retinopathy of Prematurity Among Very Low Birth Body Weight Infants. Taiwan J. Ophthalmol. 2 (2), 60–63. 10.1016/j.tjo.2012.04.001

[B45] Luo D. Wan X. Liu J. Tong T. (2018). Optimally Estimating the Sample Mean from the Sample Size, Median, Mid-range, And/or Mid-Quartile Range. Stat. Methods Med. Res. 27 (6), 1785–1805. 10.1177/0962280216669183 27683581

[B46] Lust C. Vesoulis Z. Jackups R. Liao S. Rao R. Mathur A. M. (2019). Early Red Cell Transfusion Is Associated with Development of Severe Retinopathy of Prematurity. J. Perinatol 39 (3), 393–400. 10.1038/s41372-018-0274-9 30459388 PMC6391181

[B47] Lyu J. Zhang Q. Jin H. Xu Y. Chen C. Ji X. (2018). Aqueous Cytokine Levels Associated with Severity of Type 1 Retinopathy of Prematurity and Treatment Response to Ranibizumab. Graefes Arch. Clin. Exp. Ophthalmol. 256 (8), 1469–1477. 10.1007/s00417-018-4034-5 29948178

[B48] Maini B. Chellani H. Arya S. Guliani B. P. (2014). Retinopathy of Prematurity: Risk Factors and Role of Antenatal Betamethasone in Indian Preterm Newborn Babies. J. Clin. Neonatol. 3 (1), 20–24. 10.4103/2249-4847.128724 24741536 PMC3982335

[B49] Mayock D. E. Xie Z. Comstock B. A. Heagerty P. J. Juul S. E. (2020). High-Dose Erythropoietin in Extremely Low Gestational Age Neonates Does Not Alter Risk of Retinopathy of Prematurity. Neonatology 117 (5), 1–8. 10.1159/000511262 33113526 PMC7855231

[B50] Melamed N. Shah J. Soraisham A. Yoon E. W. Lee S. K. Shah P. S. (2015). Association between Antenatal Corticosteroid Administration-To-Birth Interval and Outcomes of Preterm Neonates. Obstet. Gynecol. 125 (6), 1377–1384. 10.1097/aog.0000000000000840 26000509

[B51] Melamed N. Shah J. Yoon E. W. Pelausa E. Lee S. K. Shah P. S. (2016). The Role of Antenatal Corticosteroids in Twin Pregnancies Complicated by Preterm Birth. Am. J. Obstet. Gynecol. 215 (4), 482e481–9. 10.1016/j.ajog.2016.05.037 27260974

[B52] Moher D. Liberati A. Tetzlaff J. Altman D. G. (2009). Preferred Reporting Items for Systematic Reviews and Meta-Analyses: the PRISMA Statement. Bmj 339, b2535. 10.1136/bmj.b2535 19622551 PMC2714657

[B53] O'Connor M. T. Vohr B. R. Tucker R. Cashore W. (2003). Is Retinopathy of Prematurity Increasing Among Infants Less Than 1250 G Birth Weight? J. Perinatol 23 (8), 673–678. 10.1038/sj.jp.7211008 14647167

[B54] Ogata J. F. Fonseca M. C. de Almeida M. F. Guinsburg R. (2016). Antenatal Corticosteroids: Analytical Decision Model and Economic Analysis in a Brazilian Cohort of Preterm Infants. J. Matern. Fetal Neonatal. Med. 29 (18), 2973–2979. 10.3109/14767058.2015.1111331 26513273

[B55] Omair A. (2016). Selecting the Appropriate Study Design: Case-Control and Cohort Study Designs. J. Health Spec. 4 (1), 37. 10.4103/1658-600X.173842

[B56] Page M. J. McKenzie J. E. Bossuyt P. M. Boutron I. Hoffmann T. C. Mulrow C. D. (2021). The PRISMA 2020 Statement: An Updated Guideline for Reporting Systematic Reviews. Bmj 372, n71. 10.1136/bmj.n71 33782057 PMC8005924

[B57] Park Y. J. Woo S. J. Kim Y. M. Hong S. Lee Y. E. Park K. H. (2019). Immune and Inflammatory Proteins in Cord Blood as Predictive Biomarkers of Retinopathy of Prematurity in Preterm Infants. Invest. Ophthalmol. Vis. Sci. 60 (12), 3813–3820. 10.1167/iovs.19-27258 31525777

[B58] Perna-Barrull D. Gieras A. Rodriguez-Fernandez S. Tolosa E. Vives-Pi M. (2020). Immune System Remodelling by Prenatal Betamethasone: Effects on β-Cells and Type 1 Diabetes. Front. Endocrinol. (Lausanne) 11, 540. 10.3389/fendo.2020.00540 32849311 PMC7431597

[B59] Rathi S. Jalali S. Patnaik S. Shahulhameed S. Musada G. R. Balakrishnan D. (2017). Abnormal Complement Activation and Inflammation in the Pathogenesis of Retinopathy of Prematurity. Front. Immunol. 8, 1868. 10.3389/fimmu.2017.01868 29312345 PMC5743907

[B60] Roberts D. Brown J. Medley N. Dalziel S. R. (2017). Antenatal Corticosteroids for Accelerating Fetal Lung Maturation for Women at Risk of Preterm Birth. Cochrane Database Syst. Rev. 3, CD004454. 10.1002/14651858.CD004454.pub3 28321847 PMC6464568

[B61] Ryu Y. H. Oh S. Sohn J. Lee J. (2019). The Associations between Antenatal Corticosteroids and In-Hospital Outcomes of Preterm Singleton Appropriate for Gestational Age Neonates According to the Presence of Maternal Histologic Chorioamnionitis. Neonatology 116 (4), 369–375. 10.1159/000502650 31593959

[B62] Salhab W. A. Hynan L. S. Perlman J. M. (2003). Partial or Complete Antenatal Steroids Treatment and Neonatal Outcome in Extremely Low Birth Weight Infants. J. Perinatol. 23 (8), 668–672. 10.1038/sj.jp.7211007 14647166

[B63] Serenius F. Ewald U. Farooqi A. Holmgren P. A. Håkansson S. Sedin G. (2004). Short-Term Outcome after Active Perinatal Management at 23-25 Weeks of Gestation. A Study from Two Swedish Perinatal Centres. Part 3: Neonatal Morbidity. Acta Paediatr. 93 (8), 1090–1097. 10.1111/j.1651-2227.2004.tb02722.x 15456201

[B64] Shah V. A. Yeo C. L. Ling Y. L. Ho L. Y. (2005). Incidence, Risk Factors of Retinopathy of Prematurity Among Very Low Birth Weight Infants in Singapore. Ann. Acad. Med. Singap 34 (2), 169–178. 15827664

[B65] Smith L. M. Qureshi N. Chao C. R. (2000). Effects of Single and Multiple Courses of Antenatal Glucocorticoids in Preterm Newborns Less Than 30 Weeks' Gestation. J. Matern. Fetal Med. 9 (2), 131–135. 10.1002/(sici)1520-6661(200003/04)9:2<131:aid-mfm9>3.0.co;2-m 10902829

[B66] Smith L. E. Hard A. L. Hellström A. (2013). The Biology of Retinopathy of Prematurity: How Knowledge of Pathogenesis Guides Treatment. Clin. Perinatol 40 (2), 201–214. 10.1016/j.clp.2013.02.002 23719305 PMC3673697

[B67] Talia D. Zhu T. Agrotis A. Slattery R. Le Page M. Mackay-Fisson F. (2013). The Contribution of T Cells to Retinopathy of Prematurity in Mice. Invest. Ophthalmol. Vis. Sci. 54 (15), 2027. Abstract Retrieved From ARVO 2013 Annual Meeting Abstract. (Accession No. 628584463) 23519043

[B68] Travers C. P. Clark R. H. Spitzer A. R. Das A. Garite T. J. Carlo W. A. (2017). Exposure to Any Antenatal Corticosteroids and Outcomes in Preterm Infants by Gestational Age: Prospective Cohort Study. Bmj 356, j1039. 10.1136/bmj.j1039 28351838 PMC5373674

[B69] Tremblay S. Miloudi K. Chaychi S. Favret S. Binet F. Polosa A. (2013). Systemic Inflammation Perturbs Developmental Retinal Angiogenesis and Neuroretinal Function. Invest. Ophthalmol. Vis. Sci. 54 (13), 8125–8139. 10.1167/iovs.13-12496 24204040

[B70] van Sorge A. J. Termote J. U. Kerkhoff F. T. van Rijn L. J. Simonsz H. J. Peer P. G. (2014). Nationwide Inventory of Risk Factors for Retinopathy of Prematurity in the Netherlands. J. Pediatr. 164 (3), 494–498.e1. 10.1016/j.jpeds.2013.11.015 24360994

[B71] Villamor-Martinez E. Cavallaro G. Raffaeli G. Mohammed Rahim O. M. M. Gulden S. Ghazi A. M. T. (2018). Chorioamnionitis as a Risk Factor for Retinopathy of Prematurity: An Updated Systematic Review and Meta-Analysis. Plos One 13 (10), e0205838. 10.1371/journal.pone.0205838 30332485 PMC6192636

[B72] Vogel J. P. Souza J. P. Gülmezoglu A. M. Mori R. Lumbiganon P. Qureshi Z. (2014). Use of Antenatal Corticosteroids and Tocolytic Drugs in Preterm Births in 29 Countries: An Analysis of the WHO Multicountry Survey on Maternal and Newborn Health. Lancet 384 (9957), 1869–1877. 10.1016/s0140-6736(14)60580-8 25128271

[B73] Wang Y. C. Tseng H. I. Yang S. N. Lu C. C. Wu J. R. Dai Z. K. (2012). Effects of Antenatal Corticosteroids on Neonatal Outcomes in Very-Low-Birth-Weight Preterm Newborns: A 10-year Retrospective Study in a Medical Center. Pediatr. Neonatol. 53 (3), 178–183. 10.1016/j.pedneo.2012.04.004 22770106

[B74] Wells L. R. Papile L. A. Gardner M. O. Hartenberger C. R. Merker L. (1999). Impact of Antenatal Corticosteroid Therapy in Very Low Birth Weight Infants on Chronic Lung Disease and Other Morbidities of Prematurity. J. Perinatol. 19 (8 PART. 1), 578–581. 10.1038/sj.jp.7200268 10645523

[B75] Wells G. Shea B. O'Connell D. Peterson J. Welch V. Losos M. (2011). The Newcastle-Ottawa Scale (NOS) for Assessing the Quality of Nonrandomized Studies in Meta-Analyses. Available at: http://www.ohri.ca/programs/clinical_epidemiology/oxford.htm (Accessed August 1, 2021). 10.1016/s0022-3476(97)70031-3

[B76] Yang C. Y. Lien R. Yang P. H. Chu S. M. Hsu J. F. Fu R. H. (2011). Analysis of Incidence and Risk Factors of Retinopathy of Prematurity Among Very-Low-Birth-Weight Infants in North Taiwan. Pediatr. Neonatol. 52 (6), 321–326. 10.1016/j.pedneo.2011.08.004 22192259

[B77] Yim C. L. Tam M. Chan H. L. Tang S. M. Au S. C. L. Yip W. W. K. (2018). Association of Antenatal Steroid and Risk of Retinopathy of Prematurity: A Systematic Review and Meta-Analysis. Br. J. Ophthalmol. 102 (10), 1336–1341. 10.1136/bjophthalmol-2017-311576 29632000

[B78] Ying G. S. Bell E. F. Donohue P. Tomlinson L. A. Binenbaum G. (2019). Perinatal Risk Factors for the Retinopathy of Prematurity in Postnatal Growth and Rop Study. Ophthalmic Epidemiol. 26 (4), 270–278. 10.1080/09286586.2019.1606259 31012360

