# Supplementary material for: Beyond Fetal Immunity: A Systematic Review and Meta-Analysis of the Association Between Antenatal Corticosteroids and Retinopathy of Prematurity
Source: Front Pharmacol. 2022 Jan 28;13:759742. doi: 10.3389/fphar.2022.759742 (PMC8832004; doi:10.3389/fphar.2022.759742)
Supplement: Supplementary file 1 [file Table1.DOCX]

Supplementary Table 1. Searching Strategy

| Databases | Search | Search Strategy | Results |
| --- | --- | --- | --- |
| PubMed | #1 | ((((steroid*) OR (cortico*)) OR (betamethasone)) OR (dexamethasone)) AND (((((Retinopathy of Prematurity) OR (ROP)) OR (Prematurity Retinopathy)) OR (Retrolental Fibroplasia*)) OR (Prematurity Retinopathies)) | 343 |
| EMBASE | #1 | Retinopathy of Prematurity.mp. or retrolental fibroplasia/ | 11966 |
|  | #2 | Prematurity Retinopathy.mp. | 12 |
|  | #3 | Prematurity Retinopathies.mp. | 0 |
|  | #4 | steroid*.mp. | 452359 |
|  | #5 | cortico*.mp | 476124 |
|  | #6 | exp betamethasone/or Betamethasone.mp. | 24088 |
|  | #7 | exp dexamethasone/or dexamethasone.mp. | 171360 |
|  | #8 | 4 or 5 or 6 or 7 | 990633 |
|  | #9 | 1 or 2 or 3 | 11967 |
|  | #10 | 8 and 9 | 942 |
| Scopus | #1 | ((TITLE-ABS-KEY (retinopathy AND of AND prematurity) OR TITLE-ABS-KEY (prematurity AND retinopathy) OR TITLE-ABS-KEY (retrolental AND fibroplasia*) OR TITLE-ABS-KEY (prematurity AND retinopathies))) AND ((TITLE-ABS-KEY (steroid*) OR TITLE-ABS-KEY (cortico*) OR TITLE-ABS-KEY (betamethasone) OR TITLE-ABS-KEY (dexamethasone))) AND (LIMIT-TO (SUBJAREA, "MEDI")) | 872 |
| Web of Science | #1 | TS= (Retinopathy of Prematurity OR Prematurity Retinopathy OR ROP OR Retrolental Fibroplasia* OR Prematurity Retinopathies) AND TS= (steroid* OR cortico* OR dexamethasone OR betamethasone) | 482 |
| Cochrane Library | #1 | (Retinopathy of Prematurity): ti,ab,kw OR (ROP): ti,ab,kw OR (Prematurity Retinopathy): ti,ab,kw OR (Retrolental Fibroplasia*): ti,ab,kw OR (Prematurity Retinopathies): ti,ab,kw | 1528 |
|  | #2 | (steroid*): ti,ab,kw OR (cortico*): ti,ab,kw OR (betamethasone): ti,ab,kw OR (dexamethasone): ti,ab,kw | 63963 |
|  | #3 | #1 AND #2 | 122 |
