# Supplementary material for: Beyond Fetal Immunity: A Systematic Review and Meta-Analysis of the Association Between Antenatal Corticosteroids and Retinopathy of Prematurity
Source: Front Pharmacol. 2022 Jan 28;13:759742. doi: 10.3389/fphar.2022.759742 (PMC8832004; doi:10.3389/fphar.2022.759742)
Supplement: Supplementary file 3 [file Table2.DOCX]

Supplementary Table 2. Characteristics of all included studies

| First author, year | Country | Study design | Inclusion criteria | ACS | | Sample Size | Any ROP | Severe ROP | Adjusted Factors | NOS |
| --- | --- | --- | --- | --- | --- | --- | --- | --- | --- | --- |
|  |  |  |  | **Drug** | **Course** |  |  |  |  |  |
| Dani 2021 (Dani et al., 2021) | Italy | RCC | 23≤ GA <30w | NA | NA | 178 | uOR: 0.22 (0.11-0.43) | NA | NA | 7 |
| Taner 2020 (Taner et al., 2020) | Switzerland | RCC | GA <29w | NA | NA | 307 | aOR: 1.40 (0.24-8.01) | NA | sex, location, multiple birth, caesarean section, pulmonary hypertension, oxygen therapy, mechanical ventilation duration, BW, length of hospitalization, probability of dying | 7 |
| Opara 2020 (Opara et al., 2020) | USA | RCC | BW ≤1500g | NA | NA | 881 | NA | aOR: 0.50 (0.3–0.79) | Chorioamnionitis, maternal age, hypertension, maternal diabetes, neonatal steroids, NEC, RDS, GA, BW, BPD, IVH, sepsis, prenatal care | 7 |
| Kong 2020 (Kong et al., 2020) | China | RCS | 25≤ GA <35w | DEX | complete | 1662 | uOR: 1.47 (0.74-2.93)  aOR: 1.75 (0.82-3.70) | uOR: 0.56 (0.09-3.33) | GA, BW, gender, SGA,GDM  delivery mode, Apgar score at 5 min, PROM, HDP complicating pregnancy, placenta | 8 |
| Kim 2020 (Kim et al., 2020) | Korea | RCS | 23≤ GA <35w  BW ≤1500g | ANY | NA | 9142 | NA | uOR: 1.15 (0.94-1.42) | NA | 6 |
| Jagla 2020 (Jagla et al., 2020) | Poland | RCC | BW <1251g | NA | NA | 60 | NA | uOR: 1.12 (0.35-3.59) | NA | 6 |
| Cui 2020 (Cui, 2020) | China | PCS | GA <28w or  BW <1000g | DEX | complete/  partial | 1544 | uOR: 0.77 (0.63-0.95) | NA | NA | 9 |

Supplementary Table 2. Continued.

| First author, year | Country | | | Study design | Inclusion criteria | ACS | | Sample Size | Any ROP | Severe ROP | Adjusted Factors | NOS |
| --- | --- | --- | --- | --- | --- | --- | --- | --- | --- | --- | --- | --- |
|  |  |  |  |  |  | **Drug** | **Course** |  |  |  |  |  |
| Ying 2019 (Ying et al., 2019) | USA | | | RCC | GA <30w or  BW <1501g | NA | complete/  partial | 7483 | uOR: 0.87 (0.78-0.97) | uOR: 0.79 (0.67-0.93) | NA | 6 |
| Hand 2019 (Hand and Shrier, 2019) | USA | | | RCC | BW ≤1500g | NA | NA | 335 | uOR: 0.76 (0.24-2.40) | NA | NA | 6 |
| Ryu 2019 (Ryu et al., 2019) | Korea | | | RCS | 23< GA <34w | ANY | complete/  partial | 254 | NA | uOR: 0.45 (0.14-1.48) | NA | 7 |
| Lust 2019 (Lust et al., 2019) | USA | | | RCC | GA ≤32w or  BW <1501g | NA | NA | 1636 | NA | uOR: 0.99 (0.61-1.60) | NA | 5 |
| Chang 2019 (Chang, 2019) | | Korea | RCC | | GA ≤32w or  BW <1501g | NA | NA | 162 | uOR: 2.32 (1.37-3.93)  aOR: 5.00 (2.24-11.16) | uOR: 0.97 (0.47-2.00) | GA, Total mechanical ventilation duration, RDS | 6 |
| Wu 2018 (Wu et al., 2018) | | China | RCC | | BW <1501g | NA | NA | 507 | uOR: 1.16 (0.78-1.73) | NA | NA | 7 |
| Lynch 2018 (Lynch et al., 2018) | | USA | RCC | | GA ≤30w or  BW ≤1500g | NA | NA | 1202 | uOR: 1.73 (0.93-3.22) | uOR: 0.97 (0.34-2.78) | NA | 7 |
| Kim 2018 (Kim et al., 2018) | | Korea | RCS | | GA ≤34w | DEX | complete | 82 | uOR: 0.16 (0.013.36) | NA | NA | 7 |
| Gul 2018 (Gul and Bulbul, 2018) | | Turkey | RCC | | GA ≤34w | NA | NA | 537 | uOR: 3.09 (1.94-4.92) | NA | NA | 7 |

Supplementary Table 2. Continued.

| First author, year | Country | | Study design | Inclusion criteria | | ACS | | Sample Size | Any ROP | Severe ROP | Adjusted Factors | NOS |
| --- | --- | --- | --- | --- | --- | --- | --- | --- | --- | --- | --- | --- |
|  |  |  |  |  |  | **Drug** | **Course** |  |  |  |  |  |
| Bas 2018 (Bas et al., 2018) | Turkey | PCC | | | BW ≤1500g | NA | NA | 2417 | NA | uOR: 0.77 (0.62-0.96) | NA | 8 |
| Travers 2017 (Travers et al., 2017) | USA | RCS | | | 23< GA <35w | ANY | NA | 117941 | NA | uOR: 2.26 (1.95-2.61) | NA | 8 |
| Ali 2017 (Ali et al., 2017) | Egypt | RCC | | | GA<37w or BW<2500g | NA | NA | 92 | NA | uOR: 0.26 (0.01-4.82) | NA | 6 |
| Ogata 2016 (Ogata et al., 2016) | Brazil | RCS | | | GA ≤32w | ANY | NA | 192 | uOR: 0.50 (0.22-1.10) | uOR: 0.77 (0.08-7.17) | NA | 7 |
| Melamed 2016 (Melamed et al., 2016) | Canada | RCS | | | 24< GA <34w | ANY | complete | 9466 | NA | uOR: 1.73 (1.24-2.41)  aOR: 0.83 (0.5-1.4) | GA, sex, hypertension, outborn status, SGA, parity, and cesarean birth. | 7 |
| Maini 2014 (Maini et al., 2014) | India | PCC | | | GA ≤34w | BETA | complete | 148 | uOR: 0.61 (0.31-1.22) | uOR: 0.18 (0.02-1.48) | NA | 8 |
| Goncalves 2014 (Goncalves et al., 2014) | Brazil | PCC | | | GA≤32w or  BW≤1500g | NA | NA | 110 | uOR: 1.84 (0.73-4.66)  aOR: 1.61 (0.45-5.79) | NA | GA, BW, Apgar score, oxygen therapy, use of blood transfusion, surfactant, sepsis, BPD, phototherapy | 8 |
| van Sorge 2014 (van Sorge et al., 2014) | The  Netherlands | RCC | | | GA ≤32 w or  BW ≤1500g | NA | NA | 1380 | NA | aOR: 0.6 (0.4-0.8) | GA, BW | 7 |
| Dani 2014 (Dani et al., 2014) | Italy | RCC | | | GA<29w | NA | NA | 209 | uOR: 0.35 (0.17-0.71) | NA | NA | 6 |

Supplementary Table 2. Continued.

| First author, year | Country | Study design | Inclusion criteria | ACS | | Sample Size | Any ROP | Severe ROP | Adjusted Factors | NOS |
| --- | --- | --- | --- | --- | --- | --- | --- | --- | --- | --- |
|  |  |  |  | **Drug** | **Course** |  |  |  |  |  |
| Ahmadpour-Kacho 2014 (Ahmadpour-Kacho et al., 2014) | Iran | RCC | GA <34w or  BW <2000g | BETA | NA | 155 | uOR: 0.72 (0.35-1.45) | NA | NA | 8 |
| Sasaki 2014 (Sasaki et al., 2014) | Japan | RCS | BW ≤1500g | BETA | NA | 15765 | aOR: 0.74 (0.69-0.79) | NA | maternal age, infant gender, GA, BW, presence of twins, delivery by caesarean section, intrauterine growth retardation, PROM. | 8 |
| Woo 2013 (Woo et al., 2013) | Korea | RCC | GA<32w | NA | NA | 60 | uOR: 1.0 (0.34-2.94) | NA | NA | 8 |
| Rao 2013 (Rao et al., 2013) | India | PCC | GA ≤32 w or  BW ≤1500g | NA | NA | 282 | uOR: 1.46 (0.81-2.64) | NA | NA | 8 |
| Güran 2013 (Ömer Güran, 2013) | Turkey | RCS | GA <36w and  BW ≤1500g | NA | NA | 246 | uOR: 1.97 (1.04-3.73) | uOR: 0.49 (0.10-2.36) | NA | 6 |
| Mohamed 2013 (Ömer Güran, 2013) | USA | RCC | GA ≤32 w or  BW ≤1500g | NA | NA | 582 | uOR: 0.68 (0.39-1.20) | NA | NA | 7 |
| Wang 2012 (Wang et al., 2012) | China | RCS | BW <1500g | DEX/  BETA | NA | 256 | uOR: 1.08 (0.58-2.02) | uOR: 0.38 (0.11-1.35) | NA | 7 |
| Liu 2012 (Liu et al., 2012) | China | RCC | BW< 1500g | NA | NA | 96 | uOR: 0.08 (0.1-0.70) | uOR: 0.07 (0.01-0.62) | NA | 7 |
| Yang 2011 (Yang et al., 2011) | China | RCC | GA ≤32 w or  BW ≤1500g | BETA | NA | 216 | uOR: 0.88 (0.51-1.53) | uOR: 0.54 (0.26-1.11) | NA | 6 |
| Wikstrand 2011 (Wikstrand et al., 2011) | Sweden | RCC | GA<28w | NA | NA | 46 | NA | uOR: 0.30 (0.03-3.16) | NA | 7 |

Supplementary Table 2. Continued.

| First author, year | | Country | Study design | | | | | Inclusion criteria | ACS | | Sample Size | Any ROP | Severe ROP | Adjusted Factors | NOS |
| --- | --- | --- | --- | --- | --- | --- | --- | --- | --- | --- | --- | --- | --- | --- | --- |
|  |  |  |  |  |  |  |  |  | **Drug** | **Course** |  |  |  |  |  |
| Kumar 2011 (Kumar et al., 2011) | | India | RCC | | | | | GA ≤32 w or  BW ≤1500g | NA | complete/  partial | 645 | NA | uOR: 3.56 (1.44-8.81) | NA | 5 |
| Fortes Filho 2011 (Fortes Filho et al., 2011) | | Brazil | PCC | | | | | GA ≤32 w or  BW ≤1500g | DEX/  BETA | complete | 324 | uOR: 0.57 (0.35-0.94)  aOR: 0.56 (0.32-0.98) | uOR: 0.98 (0.42-2.29) | GA, SGA, maternal preeclampsia, hypertension, IVH, mechanical ventilation, indomethacin, blood transfusion, and vaginal delivery. | 8 |
| Chen 2011 (Chen et al., 2011) | | USA | RCC | | | | | GA< 30 w or  BW<1501g | NA | NA | 622 | uOR: 1.38 (0.82-2.33)  aOR: 1.3 (0.7–2.2) | NA | GA<26 weeks | 8 |
| Vento 2010 (Vento et al., 2010) | | Spain | PCS | | | | | GA ≤28w | BETA | complete | 57 | uOR: 0.23 (0.06-0.90) | NA | NA | 9 |
| Giapros 2011 (Giapros et al., 2011) | Greece | | | RCC | | GA ≤32 w or  BW ≤1500g | | | NA | complete | 189 | uOR: 1.08 (0.45-2.61)  aOR: 1.8 (0.4-7.7) | NA | GA, BW, sex, transportation, resuscitation, in vitro fertilization, SGA, sepsis, IVH, RDS, oxygen, PDA, chronic lung disease, PS, hospitalization (days), maternal age, HDP, 3rd trimester hemorrhage, PROM, Chorioamnionitis | 7 |
| Eriksson 2009 (Eriksson et al., 2009) | Sweden | | | RCS | | GA ≤34w | | | DEX/  BETA | complete | 7200 | uOR: 1.57 (1.03-2.37)  aOR: 0.80 (0.48-1.32) | NA | hospital level, GA, birth year | 6 |
| Dammann 2009 (Dammann et al., 2009) | Germany | | | RCC | | GA <32w | | | NA | complete | 73 | uOR: 0.54 (0.15-1.94)  aOR: 0.3 (0.1–1.2) | uOR: 0.88 (0.14-5.54) | GA | 7 |
| Al-Amro 2007 (Al-Amro et al., 2007) | | Saudi Arabia | | | PCC | | GA ≤34 w or  BW ≤2000g | | DEX | NA | 195 | NA | aOR: 0.37 (0.15–0.94) | NA | 8 |

Supplementary Table 2. Continued.

| First author, year | Country | Study design | Inclusion criteria | ACS | | Sample Size | Any ROP | Severe ROP | Adjusted Factors | NOS |
| --- | --- | --- | --- | --- | --- | --- | --- | --- | --- | --- |
|  |  |  |  | **Drug** | **Course** |  |  |  |  |  |
| Lee 2006 (Lee et al., 2006) | USA | RCS | 401g≤ BW ≤1500g | DEX/  BETA | complete | 3600 | uOR: 1.11 (0.94-1.32)  aOR: 1.13 (0.8-1.54) | uOR: 1.19 (0.88-1.62)  aOR: 1.02 (0.63-1.68) | center, maternal race, maternal marital status, prenatal care, gestational hypertension, PROM, mode of delivery, singleton birth, sex, and birth weight by 250g increments. | 8 |
| Song 2005 (Song and Han, 2005) | Korea | RCS | 24≤ GA ≤28w | DEX | complete | 39 | uOR: 0.56 (0.15-2.09) | NA | NA | 6 |
| Shah 2005 (Shah et al., 2005) | Singapore | RCC | BW ≤1500 g | BETA | complete | 564 | uOR: 1.08 (0.75-1.55) | uOR: 0.78 (0.36-1.71) | NA | 7 |
| Lubetzky 2005 (Lubetzky et al., 2005) | Israe | RCC | GA ≤28 w or  BW ≤1500g | BETA | NA | 46 | uOR: 1.00 (0.31-3.21) | NA | NA | 7 |
| Karna 2005 (Karna et al., 2005) | USA | PCC | BW ≤1500 g | BETA | complete/  partial | 576 | uOR: 0.83 (0.57-1.21)  aOR: 0.5 (0.17-1.26) | uOR: 0.83 (0.42-1.65) | GA, Race, sex, RDS, MV, PS, oxygen | 9 |
| Serenius 2004 (Serenius et al., 2004) | Sweden | RCS | 23≤ GA ≤25w | NA | complete/  partial | 140 | NA | uOR: 0.30 (0.08–1.09) | NA | 8 |
| Ng 2004 (Ng et al., 2004) | China | PCC | GA <32 w or  BW <1500g | DEX | NA | 92 | NA | uOR: 0.48 (0.16–1.42) | NA | 7 |
| O'Connor 2003 (O'Connor et al., 2003) | USA | RCC | BW <1250 g | NA | NA | 739 | uOR: 1.16 (0.83-1.63)  aOR: 0.81 (0.51-1.28) | uOR: 0.91 (0.50-1.65)  aOR: 1.20 (0.59-2.44) | NA | 7 |

Supplementary Table 2. Continued.

| First author, year | Country | Study design | Inclusion criteria | | ACS | | Sample Size | Any ROP | Severe ROP | Adjusted Factors | NOS |
| --- | --- | --- | --- | --- | --- | --- | --- | --- | --- | --- | --- |
|  |  |  |  |  | **Drug** | **Course** |  |  |  |  |  |
| Garg 2003 (Garg et al., 2003) | USA | RCC | | BW ≤1000 g | NA | NA | 47 | NA | uOR: 0.64 (0.19–2.20) | NA | 7 |
| Elimian 2003 (Elimian et al., 2003) | USA | RCS | | 23≤ GA ≤34w | BETA | partial | 229 | uOR: 1.67 (0.92–3.01) | NA | NA | 7 |
| Haroon Parupia 2001(Haroon Parupia and Dhanireddy, 2001) | USA | RCC | | BW ≤1000 g | NA | NA | 130 | NA | uOR: 0.75 (0.38–1.52) | NA | 6 |
| Seiberth 2000 (Seiberth and Linderkamp, 2000) | Germany | RCC | | BW ≤1500 g | BETA | NA | 402 | uOR: 0.53 (0.35–0.81) | NA | NA | 7 |
| Smith 2000 (Smith et al., 2000) | USA | RCS | | 24≤ GA <30w | DEX | complete | 147 | uOR: 0.43 (0.17–1.12) | NA | NA | 7 |
| Baud 1999 (Baud et al., 1999) | France | RCS | | 24≤ GA ≤31w | NA | NA | 880 | NA | uOR: 2.05 (0.41–10.22) | NA | 7 |
| Wells 1999 (Wells et al., 1999) | USA | PCS | | BW ≤1500 g | BETA | complete/  partial | 668 | NA | uOR: 1.80 (0.69–4.74) | NA | 8 |
| Higgins 1998 (Higgins et al., 1998) | USA | PCC | | BW ≤1250 g | DEX | NA | 63 | uOR: 1.10 (0.36-3.33) | uOR: 0.21 (0.02-1.98)  aOR: 0.14 (0.02-0.93) | GA, BW, RDS, BPD, PDA | 7 |

Supplementary Table 2. Continued.

| First author, year | Country | Study design | Inclusion criteria | ACS | | Sample Size | Any ROP | Severe ROP | Adjusted Factors | NOS |
| --- | --- | --- | --- | --- | --- | --- | --- | --- | --- | --- |
|  |  |  |  | **Drug** | **Course** |  |  |  |  |  |
| Console 1997 (Console et al., 1997) | Italy | RCC | GA ≤30w | DEX/  BETA | NA | 380 | uOR: 0.49 (0.27-0.89)  aOR: 0.35 (0.10-0.70) | uOR: 0.13 (0.03-0.55)  aOR: 0.07 (0.05-0.34) | GA, BW, RDS, surfactant, BPD, PDA, sex | 8 |
| Torres 1994 (Torres et al., 1994) | USA | RCS | 24≤ GA ≤33w | BETA | complete | 64 | uOR: 0.27 (0.06-1.12) | NA | NA | 7 |
| Magann 1993 (Magann et al., 1993) | USA | RCS | NA | DEX | complete | 54 | uOR: 0.32 (0.01-8.24) | NA | NA | 8 |
| Purohit 1985 (Purohit et al., 1985) | USA | RCC | BW <1750 g | NA | NA | 3025 | uOR: 0.85 (0.63-1.14) | NA | NA | 8 |

ROP: retinopathy of prematurity; ACS: antenatal corticosteroids; NOS: Newcastle-Ottawa Scale; RCC: retrospective case-control study; RCS: retrospective cohort study; PCC: prospective case-control study; PCS: prospective cohort study; GA: gestational age; BW: birth weight; DEX: Dexamethasone; BETA: Betamethasone; uOR: unadjusted OR; aOR: adjusted OR; GDM: gestational diabetes mellites; RDS: respiratory distress syndrome; BPD: bronchopulmonary dysplasia; PDA: patent ductus arteriosus; NEC: necrotizing enterocolitis; IVH: intraventricular hemorrhage; PROM: premature rupture of membranes; PS: postnatal steroids; HDP: hypertensive disease of pregnancy; SGA: small for gestational age; USA: the United States; NA: not applicable.

**REFERENCES**

Ahmadpour-Kacho, M., Jashni Motlagh, A., Rasoulinejad, S.A., Jahangir, T., Bijani, A., and Zahed Pasha, Y. (2014). Correlation Between Hyperglycemia and Retinopathy of Prematurity. *Pediatr Int* 56(5)**,** 726-730. doi: http://dx.doi.org/10.1111/ped.12371.

Al-Amro, S.A., Al-Kharfi, T.M., Thabit, A.A., and Al-Mofada, S.M. (2007). Risk Factors for Acute Retinopathy of Prematurity. *Compr Ther* 33(2)**,** 73-77. doi: 10.1007/s12019-007-8008-5.

Ali, A.A., Gomaa, N.A.S., Awadein, A.R., Al-Hayouti, H.H., and Hegazy, A.I. (2017). Retrospective Cohort Study Shows That the Risks for Retinopathy of Prematurity Included Birth Age and Weight, Medical Conditions and Treatment. *Acta Paediatrica* 106(12)**,** 1919-1927. doi: http://dx.doi.org/10.1111/apa.14019.

Bas, A.Y., Demirel, N., Koc, E., Ulubas Isik, D., Hirfanoglu, I.M., Tunc, T., et al. (2018). Incidence, Risk Factors and Severity of Retinopathy of Prematurity in Turkey (TR-ROP study): A Prospective, Multicentre Study in 69 Neonatal Intensive Care Units. *Br J Ophthalmol* 102(12)**,** 1711-1716. doi: 10.1136/bjophthalmol-2017-311789.

Baud, O., Foix-L'Helias, L., Kaminski, M., Audibert, F., Jarreau, P.H., Papiernik, E., et al. (1999). Antenatal Glucocorticoid Treatment and Cystic Periventricular Leukomalacia in Very Premature Infants. *N Engl J Med* 341(16)**,** 1190-1196. doi: 10.1056/nejm199910143411604.

Chang, J.W. (2019). Risk Factor Analysis for the Development and Progression of Retinopathy of Prematurity. *PLoS One* 14(7)**,** e0219934. doi: 10.1371/journal.pone.0219934.

Chen, M., Citil, A., McCabe, F., Leicht, K.M., Fiascone, J., Dammann, C.E.L., et al. (2011). Infection, Oxygen, and Immaturity: Interacting Risk Factors for Retinopathy of Prematurity. *Neonatology* 99(2)**,** 125-132. doi: http://dx.doi.org/10.1159/000312821.

Console, V., Gagliardi, L., De Giorgi, A., and De Ponti, E. (1997). Retinopathy of Prematurity and Antenatal Corticosteroids. The Italian ROP Study Group. *Acta Biomed Ateneo Parmense* 68 Suppl 1**,** 75-79.

Cui, Q. (2020). Antenatal Corticosteroid Administration in Extremely Preterm and Extremely Low Birth Weight Infants and Its Effects on Prognosis: A Multicentre Survey. *Chin J Perinat Med* 23(5)**,** 302-310. doi: 10.3760/cma.j.cn113903-20190823-00512.

Dammann, O., Brinkhaus, M.J., Bartels, D.B., Dordelmann, M., Dressler, F., Kerk, J., et al. (2009). Immaturity, Perinatal Inflammation, and Retinopathy of Prematurity: a Multi-Hit Hypothesis. *Early Hum Dev* 85(5)**,** 325-329. doi: 10.1016/j.earlhumdev.2008.12.010.

Dani, C., Coviello, C., Panin, F., Frosini, S., Costa, S., Purcaro, V., et al. (2021). Incidence And Risk Factors of Retinopathy of Prematurity in An Italian Cohort of Preterm Infants. *Ital J Pediatr* 47(1)**,** 64. doi: 10.1186/s13052-021-01011-w.

Dani, C., Poggi, C., Bresci, C., Corsini, I., Frosini, S., and Pratesi, S. (2014). Early Fresh-Frozen Plasma Transfusion Decreases the Risk of Retinopathy of Prematurity. *Transfusion* 54(4)**,** 1002-1007. doi: 10.1111/trf.12432.

Elimian, A., Figueroa, R., Spitzer, A.R., Ogburn, P.L., Wiencek, V., and Quirk, J.G. (2003). Antenatal Corticosteroids: Are Incomplete Courses Beneficial? *Obstet Gynecol* 102(2)**,** 352-325. doi: 10.1016/s0029-7844(03)00485-x.

Eriksson, L., Haglund, B., Ewald, U., Odlind, V., and Kieler, H. (2009). Short and Long-Term Effects of Antenatal Corticosteroids Assessed in a Cohort of 7,827 Children Born Preterm. *Acta Obstet Gynecol Scand* 88(8)**,** 933-938. doi: 10.1080/00016340903111542.

Fortes Filho, J.B., Costa, M.C., Eckert, G.U., Santos, P.G., Silveira, R.C., and Procianoy, R.S. (2011). Maternal Preeclampsia Protects Preterm Infants Against Severe Retinopathy of Prematurity. *J Pediatr* 158(3)**,** 372-376. doi: 10.1016/j.jpeds.2010.08.051.

Garg, R., Agthe, A.G., Donohue, P.K., and Lehmann, C.U. (2003). Hyperglycemia and Retinopathy of Prematurity in Very Low Birth Weight Infants. *J Perinatol* 23(3)**,** 186-194. doi: http://dx.doi.org/10.1038/sj.jp.7210879.

Giapros, V., Drougia, A., Asproudis, I., Theocharis, P., and Andronikou, S. (2011). Low Gestational Age and Chronic Lung Disease are Synergistic Risk Factors for Retinopathy of Prematurity. *Early Hum Dev* 87(10)**,** 653-657. doi: 10.1016/j.earlhumdev.2011.05.003.

Goncalves, E., Nasser, L.S., Martelli, D.R., Alkmim, I.R., Mourao, T.V., Caldeira, A.P., et al. (2014). Incidence and Risk Factors for Retinopathy of Prematurity in a Brazilian Reference Service. *Sao Paulo Medical Journal* 132(2)**,** 85-91. doi: http://dx.doi.org/10.1590/1516-3180.2014.1322544.

Gul, F., and Bulbul, A. (2018). Evaluation of Risk Factors Affecting Development of Retinopathy in Premature Infants. *J Aca Res Med* 8(3)**,** 171-176. doi: 10.5152/jarem.2018.2084.

Hand, I., and Shrier, E. (2019). Lack of Association of Intraventricular Hemorrhage with Retinopathy of Prematurity. *J Pediatr Neurol* 17(6)**,** 219-222. doi: http://dx.doi.org/10.1055/s-0038-1661344.

Haroon Parupia, M.F., and Dhanireddy, R. (2001). Association of Postnatal Dexamethasone Use and Fungal Sepsis in the Development of Severe Retinopathy of Prematurity and Progression to Laser Therapy in Extremely-Low-Birth-Weight Infants. *J Perinatol* 21(4)**,** 242-247. doi: 10.1038/sj.jp.7200531.

Higgins, R.D., Mendelsohn, A.L., DeFeo, M.J., Ucsel, R., and Hendricks-Munoz, K.D. (1998). Antenatal Dexamethasone and Decreased Severity of Retinopathy of Prematurity. *Arch Ophthalmol* 116(5)**,** 601-605. doi: 10.1001/archopht.116.5.601.

Jagla, M., Szymonska, I., Starzec, K., and Kwinta, P. (2020). Glycaemic Variability Is Associated With Treatment Requiring Retinopathy of Prematurity: A Case-Control Study. *Retina (Philadelphia, Pa.)* 41(4)**,** 711-717. doi: http://dx.doi.org/10.1097/IAE.0000000000002949.

Karna, P., Muttineni, J., Angell, L., and Karmaus, W. (2005). Retinopathy of Prematurity and Risk Factors: A Prospective Cohort Study. *BMC Pediatr* 5(1)**,** 18. doi: 10.1186/1471-2431-5-18.

Kim, J.K., Hwang, J.H., Lee, M.H., Chang, Y.S., and Park, W.S. (2020). Mortality Rate-dependent Variations in Antenatal Corticosteroid-associated Outcomes in Very Low Birth Weight Infants with 23-34 Weeks of Gestation: A Nationwide Cohort Study. *PLoS One* 15(10 October)**,** e0240168. doi: http://dx.doi.org/10.1371/journal.pone.0240168.

Kim, W.J., Han, Y.S., Ko, H.S., Park, I.Y., Shin, J.C., and Wie, J.H. (2018). Antenatal Corticosteroids and Outcomes of Preterm Small-for-Gestational-Age Neonates in a Single Medical Center. *Obstet Gynecol Sci* 61(1)**,** 7-13. doi: 10.5468/ogs.2018.61.1.7.

Kong, X., Xu, F., Wang, Z., Zhang, S., and Feng, Z. (2020). Antenatal Corticosteroids Administration on Mortality and Morbidity in Premature Twins Born at 25~34 Gestational Weeks: A Retrospective Multicenter Study. *Eur J Obstet Gynecol Reprod Biol* 253**,** 259-265. doi: http://dx.doi.org/10.1016/j.ejogrb.2020.08.003.

Kumar, P., Sankar, M.J., Deorari, A., Azad, R., Chandra, P., Agarwal, R., et al. (2011). Risk Factors for Severe Retinopathy of Prematurity in Preterm Low Birth Weight Neonates. *Indian J Pediatr* 78(7)**,** 812-816. doi: http://dx.doi.org/10.1007/s12098-011-0363-7.

Lee, B.H., Stoll, B.J., McDonald, S.A., and Higgins, R.D. (2006). Adverse Neonatal Outcomes Associated with Antenatal Dexamethasone versus Antenatal Betamethasone. *Pediatrics* 117(5)**,** 1503-1510. doi: 10.1542/peds.2005-1749.

Liu, Y.-S., Chen, T.-C., Yang, C.-H., Yang, C.-M., Huang, J.-S., Ho, T.-C., et al. (2012). Incidence, Risk Factors, and Treatment of Retinopathy of Prematurity Among Very Low Birth Body Weight Infants. *Taiwan J Ophthalmol* 2(2)**,** 60-63. doi: 10.1016/j.tjo.2012.04.001.

Lubetzky, R., Stolovitch, C., Dollberg, S., Mimouni, F.B., Salomon, M., and Mandel, D. (2005). Nucleated Red Blood Cells in Preterm Infants with Retinopathy of Prematurity. *Pediatrics* 116(5)**,** e619-e622. doi: http://dx.doi.org/10.1542/peds.2005-0915.

Lust, C., Vesoulis, Z., Jackups, R., Liao, S., Rao, R., and Mathur, A.M. (2019). Early Red Cell Transfusion is Associated with Development of Severe Retinopathy of Prematurity. *J Perinatol* 39(3)**,** 393-400. doi: http://dx.doi.org/10.1038/s41372-018-0274-9.

Lynch, A.M., Berning, A.A., Thevarajah, T.S., Wagner, B.D., Post, M.D., McCourt, E.A., et al. (2018). The Role of the Maternal and Fetal Inflammatory Response in Retinopathy of Prematurity. *Am J Reprod Immunol* 80(3)**,** e12986. doi: http://dx.doi.org/10.1111/aji.12986.

Magann, E.F., Graves, G.R., Roberts, W.E., Blake, P.G., Morrison, J.C., and Martin, J.N., Jr. (1993). Corticosteroids for Enhanced Fetal Lung Maturation in Patients With HELLP Syndrome: Impact on Neonates. *Aust N Z J Obstet Gynaecol* 33(2)**,** 131-135. doi: 10.1111/j.1479-828x.1993.tb02375.x.

Maini, B., Chellani, H., Arya, S., and Guliani, B.P. (2014). Retinopathy of Prematurity: Risk Factors and Role of Antenatal Betamethasone in Indian Preterm Newborn Babies. *J Clin Neonatol* 3(1)**,** 20-24. doi: 10.4103/2249-4847.128724.

Melamed, N., Shah, J., Yoon, E.W., Pelausa, E., Lee, S.K., Shah, P.S., et al. (2016). The Role of Antenatal Corticosteroids in Twin Pregnancies Complicated by Preterm Birth. *Am J Obstet Gynecol* 215(4)**,** 482.e481-489. doi: 10.1016/j.ajog.2016.05.037.

Ng, P.C., Kwok, A.K., Lee, C.H., Tam, B.S., Lam, C.W., Ma, K.C., et al. (2004). Early Pituitary-Adrenal Responses and Retinopathy of Prematurity In Very Low Birth Weight Infants. *Pediatr Res* 55(1)**,** 114-119. doi: 10.1203/01.pdr.0000100464.09953.c9.

O'Connor, M.T., Vohr, B.R., Tucker, R., and Cashore, W. (2003). Is Retinopathy of Prematurity Increasing Among Infants Less Than 1250 g Birth Weight? *J Perinatol* 23(8)**,** 673-678. doi: http://dx.doi.org/10.1038/sj.jp.7211008.

Ogata, J.F., Fonseca, M.C., de Almeida, M.F., and Guinsburg, R. (2016). Antenatal Corticosteroids: Analytical Decision Model and Economic Analysis in a Brazilian Cohort of Preterm Infants. *J Matern Fetal Neonatal Med* 29(18)**,** 2973-2979. doi: 10.3109/14767058.2015.1111331.

Ömer Güran, A.B., Sinan Uslu, Mesut Dursun, Umut Zubarioğlu, Asiye Nuhoğlu (2013). The Change of Morbidity and Mortality Rates in Very Low Birth Weight Infants Over Time. *Turk Pediatri Arsivi* 48**,** 102–109.

Opara, C.N., Akintorin, M., Byrd, A., Cirignani, N., Akintorin, S., and Soyemi, K. (2020). Maternal Diabetes Mellitus as an Independent Risk Factor for Clinically Significant Retinopathy of Prematurity Severity in Neonates Less Than 1500g. *PLoS One* 15(8)**,** e0236639. doi: 10.1371/journal.pone.0236639.

Purohit, D., Ellison, R., Zierler, S., Miettinen, O., and Nadas, A. (1985). Risk Factors for Retrolental Fibroplasia: Experience with 3,025 Premature Infants. National Collaborative Study on Patent Ductus Arteriosus in Premature Infants. *Pediatrics* 76(3)**,** 339-344.

Rao, K.A., Purkayastha, J., Hazarika, M., Chaitra, R., and Adith, K.M. (2013). Analysis of Prenatal and Postnatal Risk Factors of Retinopathy of Prematurity in a Tertiary Care Hospital in South India. *Indian J Ophthalmol* 61(11)**,** 640-644. doi: 10.4103/0301-4738.119347.

Ryu, Y.H., Oh, S., Sohn, J., and Lee, J. (2019). The Associations Between Antenatal Corticosteroids and In-Hospital Outcomes of Preterm Singleton Appropriate for Gestational Age Neonates according to the Presence of Maternal Histologic Chorioamnionitis. *Neonatology* 116(4)**,** 369-375. doi: http://dx.doi.org/10.1159/000502650.

Sasaki, Y., Ikeda, T., Nishimura, K., Katsuragi, S., Sengoku, K., Kusuda, S., et al. (2014). Association of Antenatal Corticosteroids and the Mode of Delivery with the Mortality and Morbidity of Infants Weighing Less Than 1,500g at Birth in Japan. *Neonatology* 106(2)**,** 81-86. doi: 10.1159/000358189.

Seiberth, V., and Linderkamp, O. (2000). Risk Factors in Retinopathy of Prematurity. A Multivariate Statistical Analysis. *Ophthalmologica* 214(2)**,** 131-135. doi: 10.1159/000027482.

Serenius, F., Ewald, U., Farooqi, A., Holmgren, P.A., Hakansson, S., and Sedin, G. (2004). Short-Term Outcome after Active Perinatal Management at 23-25 Weeks of Gestation. A study From Two Swedish Perinatal Centres. Part 3: Neonatal Morbidity. *Acta Paediatrica* 93(8)**,** 1090-1097. doi: http://dx.doi.org/10.1080/08035250410027760.

Shah, V.A., Yeo, C.L., Ling, Y.L., and Ho, L.Y. (2005). Incidence, Risk Factors of Retinopathy of Prematurity Among Very Low Birth Weight Infants in Singapore. *Ann Acad Med Singap* 34(2)**,** 169-178.

Smith, L.M., Qureshi, N., and Chao, C.R. (2000). Effects of Single and Multiple Courses of Antenatal Glucocorticoids in Preterm Newborns Less Than 30 Weeks' Gestation. *J Matern Fetal Med* 9(2)**,** 131-135. doi: 10.1002/(sici)1520-6661(200003/04)9:2<131::aid-mfm9>3.0.co;2-m.

Song, G.A., and Han, M.S. (2005). Effect of Antenatal Corticosteroid and Antibiotics in Pregnancies Complicated by Premature Rupture of Membranes Between 24 and 28 Weeks of Gestation. *J Korean Med Sci* 20(1)**,** 88-92. doi: 10.3346/jkms.2005.20.1.88.

Taner, A., Tekle, S., Hothorn, T., Adams, M., Bassler, D., and Gerth-Kahlert, C. (2020). Higher Incidence of Retinopathy of Prematurity in Extremely Preterm Infants Associated with Improved Survival Rates. *Acta Paediatrica* 109(10)**,** 2033-2039. doi: http://dx.doi.org/10.1111/apa.15197.

Torres, B.A., Groh, C.M., and Moya, F.R. (1994). Effect of Antenatal Corticosteroids and Thyrotropin-Releasing Hormones on Outcome of Surfactant-Treated Neonates. *J Matern Fetal Med* 3(6)**,** 241-245.

Travers, C.P., Clark, R.H., Spitzer, A.R., Das, A., Garite, T.J., and Carlo, W.A. (2017). Exposure to Any Antenatal Corticosteroids and Outcomes in Preterm Infants by Gestational Age: Prospective Cohort Study. *Bmj* 356**,** j1039. doi: 10.1136/bmj.j1039.

van Sorge, A.J., Termote, J.U.M., Kerkhoff, F.T., van Rijn, L.J., Simonsz, H.J., Peer, P.G.M., et al. (2014). Nationwide Inventory of Risk Factors for Retinopathy of Prematurity in the Netherlands. *J Pediatr* 164(3)**,** 494-+. doi: 10.1016/j.jpeds.2013.11.015.

Vento, M., Escrig, R., Izquierdo, I., Escobar, J., Arduini, A., Sastre, J., et al. (2010). Antenatal Steroids and Antioxidant Eenzyme Activity in Preterm Infants. *J Matern Fetal Neonatal Med* 23(SUPPL. 1)**,** 75. doi: http://dx.doi.org/10.3109/14767051003802495.

Wang, Y.C., Tseng, H.I., Yang, S.N., Lu, C.C., Wu, J.R., Dai, Z.K., et al. (2012). Effects of Antenatal Corticosteroids on Neonatal Outcomes in Very-Low-Birth-Weight Preterm Newborns: A 10-year Retrospective Study in a Medical Center. *Pediatr Neonatol* 53(3)**,** 178-183. doi: 10.1016/j.pedneo.2012.04.004.

Wells, L.R., Papile, L.A., Gardner, M.O., Hartenberger, C.R., and Merker, L. (1999). Impact of Antenatal Corticosteroid Therapy in Very Low Birth Weight Infant on Chronic Lung Disease and Other Morbidities of Prematurity. *J Perinatol* 19(8 PART. 1)**,** 578-581. doi: 10.1038/sj.jp.7200268.

Wikstrand, M.H., Hård, A.L., Niklasson, A., Smith, L., Löfqvist, C., and Hellström, A. (2011). Maternal and Neonatal Factors Associated with Poor Early Weight Gain and Later Retinopathy of Prematurity. *Acta Paediatr* 100(12)**,** 1528-1533. doi: 10.1111/j.1651-2227.2011.02394.x.

Woo, S.J., Lee, S.Y., Ahn, S.J., Ahn, J., Park, K.H., Oh, K.J., et al. (2013). The Relationship Between Cord Blood Cytokine Levels and Perinatal Factors and Retinopathy of Prematurity: A Gestational Age-Matched Case-Control study. *Invest Ophthalmol Vis Sci* 54(5)**,** 3434-3439. doi: http://dx.doi.org/10.1167/iovs.13-11837.

Wu, T., Zhang, L., Tong, Y., Qu, Y., Xia, B., and Mu, D. (2018). Retinopathy of Prematurity Among Very Low-Birth-Weight Infants in China: Incidence and Perinatal Risk Factors. *Invest Ophthalmol Vis Sci* 59(2)**,** 757-763. doi: 10.1167/iovs.17-23158.

Yang, C.Y., Lien, R., Yang, P.H., Chu, S.M., Hsu, J.F., Fu, R.H., et al. (2011). Analysis of Incidence and Risk Factors of Retinopathy of Prematurity Among Very-Low-Birth-Weight Infants in North Taiwan. *Pediatr Neonatol* 52(6)**,** 321-326. doi: 10.1016/j.pedneo.2011.08.004.

Ying, G.S., Bell, E.F., Donohue, P., Tomlinson, L.A., and Binenbaum, G. (2019). Perinatal Risk Factors for the Retinopathy of Prematurity in Postnatal Growth and Rop Study. *Ophthalmic Epidemiol* 26(4)**,** 270-278. doi: http://dx.doi.org/10.1080/09286586.2019.1606259.
