# Supplementary material for: Beyond Fetal Immunity: A Systematic Review and Meta-Analysis of the Association Between Antenatal Corticosteroids and Retinopathy of Prematurity
Source: Front Pharmacol. 2022 Jan 28;13:759742. doi: 10.3389/fphar.2022.759742 (PMC8832004; doi:10.3389/fphar.2022.759742)
Supplement: Supplementary file 5 [file Table3.DOCX]

Supplementary Table 3. Assessment of risk of bias through the Newcastle-Ottawa Scale

| Author, year | Selection (4) | Comparability  (2) | Outcome  (3) | Total (9) |
| --- | --- | --- | --- | --- |
| Dani 2021 | 4 | 0 | 3 | 7 |
| Taner 2020 | 3 | 1 | 3 | 7 |
| Opara 2020 | 4 | 2 | 1 | 7 |
| Kong 2020 | 3 | 2 | 3 | 8 |
| Kim 2020 | 4 | 0 | 2 | 6 |
| Jagla 2020 | 3 | 2 | 1 | 6 |
| Cui 2020 | 4 | 2 | 3 | 9 |
| Hand 2019 | 3 | 0 | 3 | 6 |
| Ryu 2019 | 4 | 1 | 2 | 7 |
| Lust 2019 | 3 | 0 | 2 | 5 |
| Chang 2019 | 2 | 2 | 2 | 6 |
| Wu 2018 | 4 | 1 | 2 | 7 |
| Lynch 2018 | 4 | 0 | 3 | 7 |
| Kim 2018 | 4 | 0 | 3 | 7 |
| Gul 2018 | 4 | 0 | 3 | 7 |
| Bas 2018 | 4 | 1 | 3 | 8 |
| Travers 2017 | 4 | 1 | 3 | 8 |
| Ali 2017 | 3 | 0 | 3 | 6 |
| Ogata 2016 | 4 | 1 | 2 | 7 |
| Melamed 2016 | 4 | 0 | 3 | 7 |
| Maini 2014 | 4 | 1 | 3 | 8 |
| Goncalves 2014 | 4 | 1 | 3 | 8 |
| van Sorge 2014 | 4 | 1 | 2 | 7 |
| Dani 2014 | 4 | 0 | 2 | 6 |
| Sasaki 2014 | 4 | 2 | 2 | 8 |
| Ahmadpour-Kacho 2014 | 4 | 1 | 3 | 8 |
| Woo 2013 | 4 | 1 | 3 | 8 |
| Rao 2013 | 4 | 1 | 3 | 8 |
| Güran 2013 | 3 | 1 | 2 | 6 |
| Mohamed 2013 | 4 | 0 | 3 | 7 |
| Wang 2012 | 3 | 1 | 3 | 7 |
| Liu 2012 | 4 | 0 | 3 | 7 |
| Yang 2011 | 4 | 0 | 2 | 6 |
| Wikstrand 2011 | 3 | 1 | 3 | 7 |
| Kumar 2011 | 3 | 0 | 2 | 5 |
| Fortes Filho 2011 | 4 | 1 | 3 | 8 |
| Chen 2011 | 4 | 1 | 3 | 8 |
| Giapros 2011 | 4 | 0 | 3 | 7 |
| Vento 2010 | 4 | 2 | 3 | 9 |
| Eriksson 2009 | 4 | 1 | 1 | 6 |
| Dammann 2009 | 3 | 1 | 3 | 7 |
| AL-AMRO 2007 | 4 | 2 | 2 | 8 |
| Lee 2006 | 4 | 1 | 3 | 8 |
| Song 2005 | 3 | 1 | 2 | 6 |

Supplementary Table 3. Continued.

| Author, year | Selection (4) | Comparability  (2) | Outcome  (3) | Total (9) |
| --- | --- | --- | --- | --- |
| Shah 2005 | 4 | 0 | 3 | 7 |
| Lubetzky 2005 | 4 | 1 | 2 | 7 |
| Karna 2005 | 4 | 2 | 3 | 9 |
| Serenius 2004 | 4 | 1 | 3 | 8 |
| Ng 2004 | 3 | 1 | 3 | 7 |
| O'Connor 2003 | 4 | 0 | 3 | 7 |
| Garg 2003 | 3 | 1 | 3 | 7 |
| Elimian 2003 | 4 | 0 | 3 | 7 |
| Haroon Parupia 2001 | 3 | 0 | 3 | 6 |
| Seiberth 2000 | 4 | 1 | 2 | 7 |
| Smith 2000 | 3 | 2 | 1 | 7 |
| Baud 1999 | 4 | 0 | 3 | 7 |
| Wells 1999 | 4 | 2 | 2 | 8 |
| Higgins 1998 | 4 | 0 | 3 | 7 |
| Console 1997 | 4 | 1 | 3 | 8 |
| Torres 1994 | 3 | 2 | 2 | 7 |
| Magann 1993 | 3 | 2 | 3 | 8 |
| Purohit 1985 | 4 | 1 | 3 | 8 |
