# Supplementary material for: Beyond Fetal Immunity: A Systematic Review and Meta-Analysis of the Association Between Antenatal Corticosteroids and Retinopathy of Prematurity
Source: Front Pharmacol. 2022 Jan 28;13:759742. doi: 10.3389/fphar.2022.759742 (PMC8832004; doi:10.3389/fphar.2022.759742)
Supplement: Supplementary file 6 [file Table4.DOCX]

Supplementary Table 4. Meta-regression analysis of antenatal corticosteroids exposure and risk of retinopathy of prematurity based on unadjusted data

| ROP Type | Covariates | k | Coefficient | 95% CI | | P | Adjusted R^2^ |
| --- | --- | --- | --- | --- | --- | --- | --- |
|  |  |  |  | **Lower limit** | **Upper limit** |  |  |
| Any ROP | Sample size, n | 41 | 0.00 | 0.00 | 0.00 | 0.31 | -3.62 |
|  | Study design (case-control/cohort) | 41 | 0.01 | -0.43 | 0.44 | 0.98 | -4.55 |
|  | GA of cohort, wk | 36 | -0.23 | -0.01 | 0.25 | 0.07 | 9.9 |
|  | BW of cohort, g | 37 | 0.00 | 0.00 | 0.00 | **0.02** | 20.08 |
|  | Male, % | 31 | 0.00 | -0.06 | 0.06 | 0.99 | -6.07 |
|  | Multiple pregnancy, % | 17 | 0.00 | -0.02 | 0.01 | 0.69 | -6.45 |
|  | RDS, % | 22 | 0.00 | -0.01 | 0.01 | 0.71 | -16.54 |
|  | MV, % | 14 | 0.01 | -0.01 | 0.03 | 0.26 | 2.55 |
|  | Surfactant, % | 17 | 0.00 | -0.01 | 0.02 | 0.80 | -8.97 |
|  | PDA, % | 25 | -0.02 | -0.03 | 0.00 | **0.02** | 30.77 |
|  | Sepsis, % | 24 | 0.00 | -0.02 | 0.01 | 0.97 | -5.85 |
|  | BPD, % | 15 | -0.01 | -0.03 | 0.01 | 0.29 | 2.08 |
|  | NEC, % | 18 | 0.06 | -0.02 | 0.14 | 0.12 | 14.93 |
|  | IVH grade III/IV, % | 15 | -0.04 | -0.10 | 0.02 | 0.15 | 0.95 |
|  | Mortality, % | 13 | 0.00 | -0.05 | 0.05 | 0.91 | -13.99 |
| Severe ROP | Sample size, n | 35 | 0.00 | 0.00 | 0.00 | **0.003** | 58.21 |
|  | Study design (case-control/cohort) | 35 | 0.47 | 0.06 | 0.87 | **0.03** | 48.88 |
|  | GA of cohort, wk | 18 | 0.15 | -0.05 | 0.34 | 0.13 | 20.97 |
|  | BW of cohort, g | 19 | 0.00 | 0.00 | 0.00 | 0.14 | 41.01 |
|  | Male, % | 23 | 0.01 | -0.07 | 0.09 | 0.73 | -1.26 |
|  | Multiple pregnancy, % | 14 | 0.00 | -0.02 | 0.03 | 0.80 | -2.88 |
|  | PDA, % | 12 | -0.01 | -0.03 | 0.01 | 0.31 | -29.18 |
|  | Sepsis, % | 17 | 0.00 | -0.02 | 0.01 | 0.54 | 1.45 |
|  | IVH grade III/IV, % | 11 | -0.04 | -0.09 | 0.02 | 0.16 | 22.17 |

ROP: retinopathy of prematurity; RDS: respiratory distress syndrome; MV: mechanical ventilation; PDA: patent ductus arteriosus; BPD: bronchopulmonary dysplasia; IVH: intraventricular hemorrhage; wk: week; CI: confidential interval; k: number of studies
